# A Benchtop Fractionation Procedure for Subcellular Analysis of the Plant Metabolome

**DOI:** 10.3389/fpls.2016.01912

**Published:** 2016-12-22

**Authors:** Lisa Fürtauer, Wolfram Weckwerth, Thomas Nägele

**Affiliations:** ^1^Department of Ecogenomics and Systems Biology, University of ViennaVienna, Austria; ^2^Vienna Metabolomics Center, University of ViennaVienna, Austria

**Keywords:** subcellular analysis, compartmentalization, non-aqueous fractionation, metabolome, cold acclimation, *Arabidopsis*

## Abstract

Although compartmentation is a key feature of eukaryotic cells, biological research is frequently limited by methods allowing for the comprehensive subcellular resolution of the metabolome. It has been widely accepted that such a resolution would be necessary in order to approximate cellular biochemistry and metabolic regulation, yet technical challenges still limit both the reproducible subcellular fractionation and the sample throughput being necessary for a statistically robust analysis. Here, we present a method and a detailed protocol which is based on the non-aqueous fractionation technique enabling the assignment of metabolites to their subcellular localization. The presented benchtop method aims at unraveling subcellular metabolome dynamics in a precise and statistically robust manner using a relatively small amount of tissue material. The method is based on the separation of cellular fractions via density gradients consisting of organic, non-aqueous solvents. By determining the relative distribution of compartment-specific marker enzymes together with metabolite profiles over the density gradient it is possible to estimate compartment-specific metabolite concentrations by correlation. To support this correlation analysis, a spreadsheet is provided executing a calculation algorithm to determine the distribution of metabolites over subcellular compartments. The calculation algorithm performs correlation of marker enzyme activity and metabolite abundance accounting for technical errors, reproducibility and the resulting error propagation. The method was developed, tested and validated in three natural accessions of *Arabidopsis thaliana* showing different ability to acclimate to low temperature. Particularly, amino acids were strongly shuffled between subcellular compartments in a cold-sensitive accession while a cold-tolerant accession was characterized by a stable subcellular metabolic homeostasis. Finally, we conclude that subcellular metabolome analysis is essential to unambiguously unravel regulatory strategies being involved in plant-environment interactions.

## Introduction

**Figure 1:**
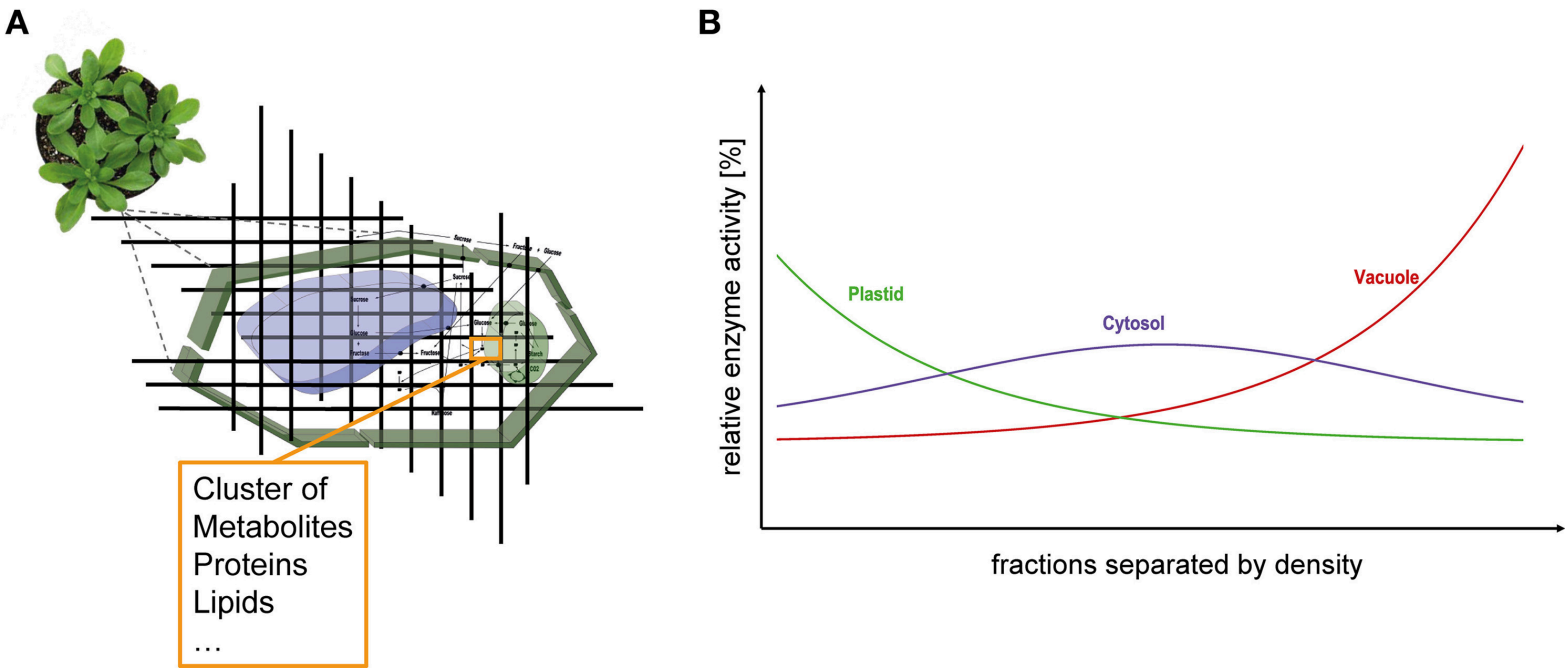
**Subcellular analysis of plant leaf tissue. (A)** Schematic representation of the dissection of leaf cells. Fractions have specific densities according to their composition of various components (e.g., proteins, metabolites, lipids etc.) and can be separated within a density gradient. **(B)** Ideal distribution of the compartments plastid (green), cytosol (purple), and vacuole (red) separated according to their densities.

Eukaryotic cells are characterized by a high degree of compartmentalization establishing a variety of biochemical reaction conditions. In particular, plant cells possess one of the most compartmentalized cell structures across all kingdoms of life. Thus, although true for all eukaryotes, particularly metabolism of plant cells is challenging to analyse due to the high diversity of metabolic pathways (Lunn, 2007). The interconnection of subcellular compartments by various transport and shuttle systems enables a regulated exchange of metabolites across biological membrane systems (Linka and Weber, 2010). A limiting step to unravel such a non-intuitive metabolic system is the lack of knowledge about distribution and dynamics of metabolite concentrations and enzyme activities (Masakapalli et al., 2010; Nägele, 2014). These data are essential for modeling approaches (Nägele, 2014) but also for biotechnological applications, e.g., metabolic engineering (Mintz-Oron et al., 2012). To gain knowledge about compartment specific metabolite levels, Gerhardt and Heldt developed the so-called non-aqueous fractionation (NAF) technique and applied it, e.g., in spinach leaves (Gerhardt and Heldt, 1984) where a density gradient allows the separation of plastids, cytosol and vacuole. Although this technique is time consuming and technically challenging (Stitt et al., 1989; Geigenberger et al., 2011) it is still the method of choice for the assignment of subcellular location of metabolites (Arrivault et al., 2014). The combination of subcellular compartmentation with experimental high-throughput analyses, e.g., mass spectrometry-coupled chromatography techniques, is deemed to be an important part of current and future biological research (Kueger et al., 2012; Tiessen and Padilla-Chacon, 2012). The basic principle of the NAF technique is the subdivision of a cell in small fractions comprising metabolites, lipids, proteins, enzymes, and all other cellular components (Figure 1A). These fractions are derived from a snap-freeze procedure of the biological material, typically using liquid nitrogen, followed by the lyophilization of a finely ground powder of the material. With this, all metabolic reactions are immediately stopped and the composition of the fractions remains unaltered due to the loss of reaction solvent. Consequently, quenched marker enzymes cluster together with metabolites, lipids and proteins finally resulting in a compartment-specific fraction density (Figure 1B). Typically, vacuolar fractions of plant leaf material possess the highest density followed by cytosolic fragments and plastidial fractions comprising lipid-rich thylakoids. Finally, these fractions are separated in a non-aqueous density gradient via ultracentrifugation. The gradient with the different fractions is then evaluated measuring marker enzyme activities and correlating the relative compound abundances with the activity distribution (Gerhardt and Heldt, 1984; Geigenberger et al., 2011; Nägele and Heyer, 2013; Arrivault et al., 2014).

Although the NAF procedure represents a comprehensive and powerful technique, its application is technically challenging frequently affecting the reproducibility and statistical robustness of the output. Additionally, ultracentrifugation steps over hours are necessary which dramatically limit the throughput capacity of the method.

To overcome these limitations and to ease the accessibility of subcellular metabolite distributions, we re-designed the experimental workflow finally leading to a protocol which enables the fractionation procedure on-the-bench. Additionally, we developed an algorithm to correlate marker enzyme activities and other compounds of interest accounting for technical error propagation. We confirmed our method in the genetic model plant *Arabidopsis thaliana* and provide new insights into the stress-induced subcellular re-organization of the primary metabolome.

## A benchtop fractionation procedure

**Figure 2:**
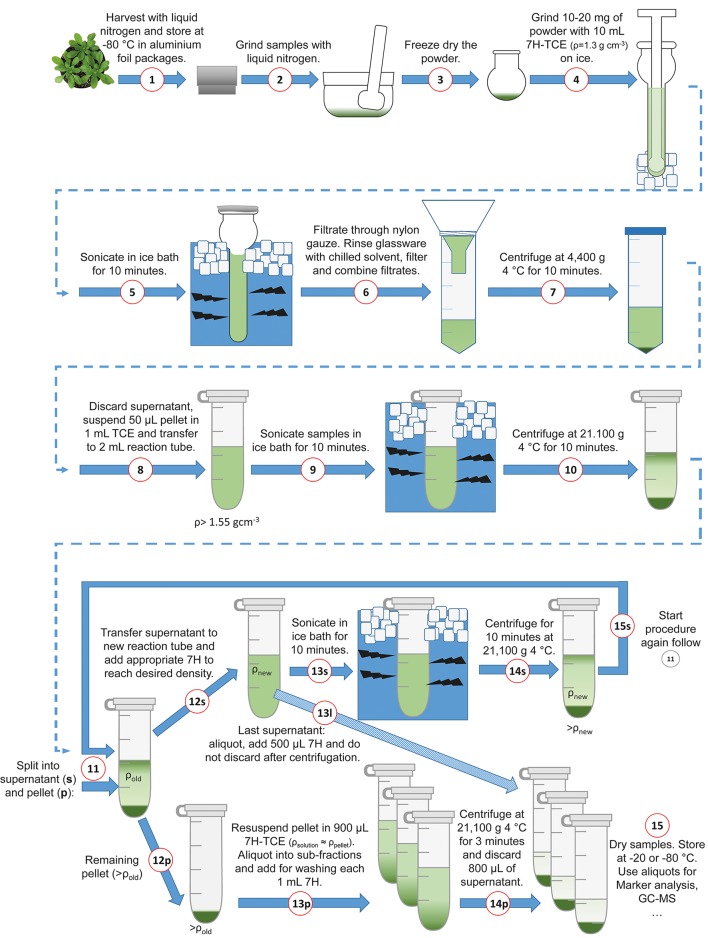
**Schematic overview of the benchtop fractionation procedure**. Harvesting, grinding of leaf material and sonication (Steps **1–5**); Filtration, collection of material and separation of fractions (Steps **6–9**); Centrifugation (Step **10**); Iterative supernatant (“s”) workflow with new density, sonication and centrifugation (Steps **12–15s**); Break-off from the supernatant loop (Step **13l**); Pellet (“p”) resuspension, generation of aliquots and washing (Steps **12–24p**); Sample drying (Step **15**). **7H**, n-Heptane; TCE, Tetrachlorethylene.

The following section comprises a detailed description of the benchtop fractionation protocol. To support the application, the fractionation procedure is illustrated in Figure 2. In a first step, approximately 10 to 20 mg of finely ground and lyophilized leaf material was suspended in 10 mL of a pre-chilled (4°C) heptane (C_7_H_16_; “7H”)-tetrachlorethylene (C_2_Cl_4_; “TCE”)-mixture with a density of ρ = 1.3 g cm^−3^(ratio 7H:TCE = 0.484:1). In a second step, the suspended plant material was ground on ice in a 15 mL glass tissue grinder with a tight mortar (Dounce Tissue Grinder, Wheaton USA) in order to further reduce the average particle size of the plant material (Figure 2; Steps **1–4)**. After homogenization, the glass vials were placed in an ice water filled ultrasonic bath (Bandelin Sonorex, Typ RK 100, 50/60 Hz), and sonicated for 10 min. Every 2 min, samples were checked to assure that they remained ice-cold. After sonication, the average particle size was in the range of nanometers to low micrometers (Supplementary Image 1). The sonicated suspension was filtered through 22–25 μm pore nylon gauze (Miracloth, Calbiochem) into a 50 mL reaction tube. The glass grinder was rinsed with 10 mL of prechilled heptane which was then filtered through the gauze and pooled with the filtered sample. The pooled filtered suspension was centrifuged with 4400 g at 4°C for 10 min (Figure 2; Steps **5–7**). The supernatant was discarded as efficient as possible and the remaining pellet, still containing approximately 50 μL of the supernatant, was re-suspended in 1 mL of tetrachlorethylene and transferred to a 2 mL reaction tube (Figure 2; Step **8**). The estimation of the supernatant remaining in the pellet was performed using a pipette and is necessary to reliably approximate the first gradient density (fraction “f,” indicated as ρ > 1.55). The samples were sonicated for 10 min in an ice water ultrasonic bath, and afterwards centrifuged in a benchtop microcentrifuge with 21,100 g at 4°C for 10 min (Figure 2; Steps **9–11**; pictures in Supplementary Images 1, 2). After centrifugation, there was a split into supernatant (“s”) and pellet (“p”). In step **12s** (Figure 2), the supernatant was transferred to a new 2 mL reaction tube and diluted with heptane according to the next gradient step. The amount of the added heptane depended on the next desired density and was calculated as described in Equation (1). The formula contains the sum of mass concentrations of *i* components of a solution divided by the total volume:

(1) $$\begin{array}{l} \rho_{solution}=\frac{1}{V_{total}}\sum_{i}m_{i}=\frac{\sum_{i}\rho_{i}V_{i}}{V_{total}}, \end{array}$$

Here, *m*_*i*_ represents the mass, *V*_*i*_ the volume and *V*_*total*_ the total volume of solution.

For example, given a supernatant volume of 1.050 mL with a density of 1.55 g cm^−3^ and a desired density of 1.45 g cm^−3^ in the next centrifugation step, inserting all volumes and densities in Equation (1) reveals the volume *x* of heptane (ρ = 0.68 g cm^−3^) which has to be added:

$$\begin{array}{l} 1.45\text{g c}{\text{m}}^{-3}=\frac{1.55\text{g c}{\text{m}}^{-3}\cdot 1.05\text{mL}+0.68\text{g c}{\text{m}}^{-3}\cdot x\text{ mL}}{1.05\text{mL}+x\text{ mL}} \end{array}$$

Solving for *x* reveals *x* = 0.137 mL, i.e., 0.137 mL of heptane has to be added to the supernatant.

The reaction tube with the new density was again sonicated in an ice bath for 10 min and afterwards centrifuged with 21,100 g at 4°C for 10 min (Figure 2, Steps **13–14s**; pictures in Supplementary Image 2). Then, the iterative procedure started again for the second fraction and continued until the final density was reached (Figure 2, Step **15s**). Examples for different density gradients are provided in the supplements (Supplementary Data 1). The remaining pellets after Step 12s were dissolved in 900 μL heptane-tetrachlorethylene according to the density of the corresponding fraction and were aliquoted into three sub-fractions. Those three sub-fractions were washed with 1 mL heptane and centrifuged for 3 min with 21,100 g at 4°C. Afterwards, 800 μL of the supernatant were discarded (Figure 2, Steps **12–14p**). The supernatant of the last fraction “l” (fraction number 1) was aliquoted into sub-fractions, e.g., 3, and 500 μL heptane were added, centrifuged at 21,100 g 4°C for 3 min and the supernatant was kept (Figure 2, Step **13l**). Samples were dried in a vacuum concentrator (LaboGene™, Denmark) and stored at −20°C until use for enzyme activity measurement and metabolome analysis (Figure 2, Step **15**).

Following the fractionation procedure, marker enzyme activities were determined photometrically, i.e., activity of plastidial pyrophosphatase activity, cytosolic uridine 5′-diphosphoglucose pyrophosphorylase and vacuolar acidic phosphatase. Finally, the relative distribution of marker enzyme activities for plastid, cytosol, and vacuole was determined which allowed for the correlation with metabolite levels.

At this point we would like to make some general technical remarks which we think are of central importance for a successful workflow: all used solvents have been pre-chilled, the work was always performed on ice and under the fume hood and special attention has to be given that samples are not contaminated with water. All open glass grinders were covered with aluminum foil when they were not used. The design and application of density gradients might differ significantly between species, organs, tissues and cell types. While it is hardly predictable which gradient has to be applied to an uncharacterized tissue or cell type, we recommend to begin with a linear gradient covering a large density range, e.g., a gradient ranging from 1.2 to 1.55 g cm^−3^ (for detailed example gradients, please refer to Supplementary Data 1). Results of the *first* enzyme marker measurements will then indicate whether the chosen linear gradient is suitable for a clear separation or whether more density steps are necessary to resolve a certain density range.

## Calculation algorithm for the determination of subcellular metabolite distributions

**Figure 3:**
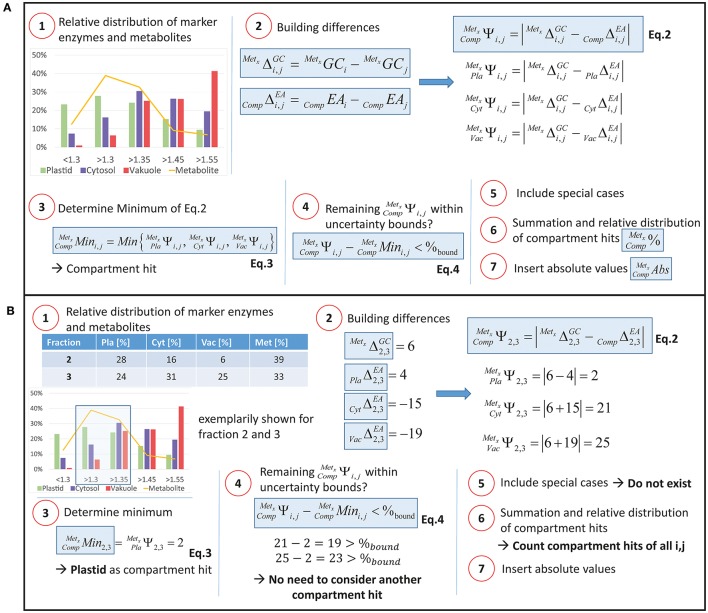
**Calculation algorithm. (A)** General overview of the algorithm. A detailed description of every step is provided in Supplementary Data 2. **(B)** Example for two fractions and one metabolite for steps **1–5**. Met_x_, Metabolite x; Comp, Compartment; Pla, Plastid; Cyt, Cytosol; Vac, Vacuole.

The relative distribution of marker enzyme activities (EA) was assumed to represent the relative distribution of corresponding subcellular compartments across density gradients. To correlate the relative distribution of marker enzyme activities with relative metabolite abundance, changes of both enzyme activities and metabolite abundance were compared pairwise between all measured fractions of one sample. The algorithm is described in detailed steps in the supplements (Supplementary Data 2) and is exemplarily shown in Figure 3. In brief, extraction and measurements resulted in *m* Metabolites (*x* = {1, …, m}) and *f* fractions (*i, j* = {1, …, *f*} *and i* < *j*) for each sample. Slopes between two fractions were built for all marker enzymes and metabolites, resulting in $\left(\begin{array}{c} f\\ 2 \end{array}\right)$ possibilities for every compartment (${Comp}^{\Delta_{i,j}^{EA}}$, Figure 3, Step **2**) and every metabolite (*^Met_x_^*$\Delta_{i,j}^{GC}$, Figure 3, Step **2**). With this, the absolute ***difference***, i.e., distance, of every metabolite to the compartment was determined, ΨCompMetxi,j (Equation 2):

(2) $$\begin{array}{l} {}_{Pla}^{Met_{x}}\Psi_{i,j}={|}^{Met_{x}}\Delta_{i,j}^{GC}-{\;}_{Pla}\Delta_{i,j}^{EA}|\\ {}_{Cyt}^{Met_{x}}\Psi_{i,j}={|}^{Met_{x}}\Delta_{i,j}^{GC}-{\;}_{Cyt}\Delta_{i,j}^{EA}|\\ {}_{Vac}^{Met_{x}}\Psi_{i,j}={|}^{Met_{x}}\Delta_{i,j}^{GC}-{\;}_{Vac}\Delta_{i,j}^{EA}| \end{array}$$

Then, the ***minimum*** of Equation (2) was built, ${}_{Comp}^{{Met}_{x}}{\text{Min}}_{i,j}$ (Equation 3):

(3) $$\begin{array}{l} {}_{Comp}^{Met_{x}}{\text{Min}}_{i,j}=Min\left\{{}_{Pla}^{Met_{x}}\Psi_{i,j},{\;}_{Cyt}^{Met_{x}}\Psi_{i,j},{\;}_{Vac}^{Met_{x}}\Psi_{i,j}\right\}, \end{array}$$

The minimum was counted as a ***hit*** for the metabolite within the respective compartment (see Figure 3A, Step **3**).

Afterwards, all other differences between slopes of metabolites and compartments, i.e., ${}_{Comp}^{{Met}_{x}}\Psi_{i,j}$, were subtracted from the solution of Equation (3) (${}_{Comp}^{{Met}_{x}}{\text{Min}}_{i,j}$) (see Figure 3, **Step4**). Finally, these differences were compared to pre-defined (uncertainty) ***bounds*** of 5, 7.5, and 10% introducing a methodological standard error in the slope interpretation (Equation 4).

(4) $$\begin{array}{l} {}_{Comp}^{Met_{x}}\Psi_{i,j}{-}_{Comp}^{Met_{x}}{\text{Min}}_{i,j}<{\%}_{bound} \end{array}$$

These bounds were chosen according to the (average) technical errors of photometric marker enzyme activity measurements and metabolite quantification using gas chromatography coupled to mass spectrometry (data not shown).

If inequation (Equation 4) was true, the corresponding compartment was additionally counted as a compartment hit. By including three of these error bounds (5, 7.5, and 10%) instead of only one error bound (e.g., 10%) slight differences between slopes could be differentiated from strong differences because, depending on the extend of the error bound, a different number of hits was assigned to metabolites.

A ***special case*** was assumed when a metabolite was only detected in a single fraction (Figure 3, Step **5**). Then, only the compartment with the highest marker enzyme activity in this fraction was taken into account.

Finally, after all these steps, all hits from the compartments were summed up for each metabolite and a ***relative distribution*** was built. The arithmetical mean and standard deviation for every metabolite and compartment ${}_{Comp}^{{Met}_{x}}\%$ (Figure 3, Step **6**) was determined. To estimate ***absolute subcellular metabolite levels***, values for relative metabolite distribution were multiplied with absolute metabolite levels derived from non-fractionated samples (${}_{Comp}^{{Met}_{x}}Abs$; Figure 3, Step **7**). A fill-in form for the calculation algorithm is provided in the supplements (Supplementary Data 3).

## Results

### Resolving the relative subcellular distribution of the primary metabolome

**Figure 4:**
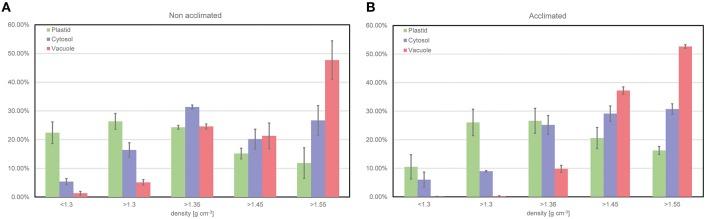
**Relative distribution of compartment-specific marker enzyme activities. (A)** Distribution resolved for non-acclimated plant leaf material of Col-0 (*n* = 3, MW ± SE), **(B)** Distribution resolved for acclimated plant leaf material of Col-0 (*n* = 3, MW ± SE). Plastid (green), Cytosol (purple) and Vacuole (red).

To demonstrate the output of the fractionation procedure, leaf material of the *Arabidopsis* accession Col-0 was analyzed combining the NAF method with GC-MS analysis of the primary metabolome. The subcellular distribution of the primary leaf metabolome in Col has been analyzed in previous studies (see e.g., Szecowka et al., 2013; Arrivault et al., 2014), and, hence, represents a suitable model tissue for new methodological development. In addition to plants grown under ambient conditions (22°C), we also analyzed leaf material of cold acclimated plants (7 days at 5°C) in order to confirm the capability of the method to resolve stress-induced metabolic reprogramming (Hoermiller et al., 2016). Activities of marker enzymes for the three compartments chloroplast, cytosol, and vacuole showed a characteristically different trend across all fractions being a prerequisite for a reliable correlation with associated metabolite levels (Figure 4). Three replicates were found to be sufficient to yield a reproducible and representative mean value for each marker enzyme activity (Figure 4). In summary, this provided evidence for a reproducible procedure for plant leaf tissue under control and stress conditions.

To reveal the subcellular metabolome distribution, metabolite levels were correlated with the marker enzyme distribution applying the calculation algorithm described in the previous section. This resulted in the relative distribution of metabolites per compartment, ${}_{Comp}^{Met_{x}}\%$ (Table 1), comprising sugars, sugar alcohols, polyamines, organic acids and amino acids. Comparing means of glucose and fructose the distribution indicated a similar trend among compartments under both conditions. Comparing the relative distribution under both conditions revealed a shift of glucose, fructose, melibiose, and threitol from the vacuolar part into the chloroplast. Additionally, melibiose showed a relative increase in the cytosolic fraction. Significant shifts were observed for the relative distribution of sucrose, raffinose, and galactinol, predominantly from the cytosol into the vacuole. For maltose and myo-inositol we observed a cold-induced shift from the chloroplast into the vacuole while the cytosolic fraction remained similar between the two conditions. The polyamine spermidine was significantly shifted from the plastidial compartment into the vacuole, while for putrescine the relative fraction in chloroplasts and cytosol decreased and the vacuolar fraction significantly increased. Under both conditions, most of the relative amount of organic acids was found to be located in the vacuole, while oxaloacetate and 2-oxoglutarate were rather located in the cytosol. Particularly, the TCA cycle intermediates citric acid, fumarate, malate, and succinate were found to be distributed similarly. Pyruvate was shifted from the vacuole into the plastid during cold acclimation, and threonate showed a significant shift into the vacuole.

**Table 1:** **Relative distribution of metabolites in Col-0 before and after cold acclimation**.

		**Col-0, Relative Distribution: *Mean* ± *SD* [%]**								
		**CHLOROPLAST**			**CYTOSOL**			**VACUOLE**		
**METABOLITES**		**Non Acc**	**Acc**	***p***	**Non Acc**	**Acc**	***p***	**Non Acc**	**Acc**	***p***
SUGARS/SUGAR ALCOHOLS	Fructose	17.2 ± 5.7	22.6 ± 4.5		29.6 ± 10.4	28.5 ± 3.7		53.2 ± 14.5	48.9 ± 5.3	
	Galactinol	27.7 ± 8	22.1 ± 6.7		45.3 ± 8.4	35.7 ± 5.5	↓^*^	27 ± 11.9	42.2 ± 6.3	↑^**^
	Glucose	18.1 ± 4.1	23.7 ± 4.7	↑^*^	28.9 ± 8.3	28.1 ± 3.7		53 ± 10.4	48.3 ± 6.2	
	Melibiose	23.5 ± 15.4	38.2 ± 5	↑^*^	21.8 ± 9.4	35.5 ± 2.8	↑^**^	47.2 ± 32	26.3 ± 7	
	myo-Inositol	40.7 ± 4.8	32.5 ± 6	↓^*^	38.1 ± 2.6	39.1 ± 5		17 ± 2.4	28.5 ± 2.8	↑^***^
	Raffinose	25.4 ± 6.5	19.7 ± 8.2		43.9 ± 9.3	32.7 ± 4.4	↓^*^	26.8 ± 5	43.6 ± 6.5	↑^**^
	Sucrose	29.7 ± 5.2	27.6 ± 5.2		47.2 ± 2.1	39.4 ± 3.2	↓^***^	24.6 ± 6.1	33 ± 3.3	↑^**^
	Threitol	19.9 ± 14.5	23 ± 14.4		27.5 ± 7.7	29.4 ± 15.4		53.8 ± 20.8	48.5 ± 26	
ORGANIC ACIDS	2-Oxoglutarate	32.3 ± 1.8	26.4 ± 14.6		47.9 ± 9.1	39.3 ± 7.4		25.5 ± 2.8	25.1 ± 12.7	
	Citrate	21.2 ± 10.3	20.3 ± 7.8		32.3 ± 10.1	31.9 ± 5.1		46.5 ± 12.3	47.8 ± 9.8	
	Fumarate	19.1 ± 13.7	19.3 ± 6		28.7 ± 11.3	28.4 ± 5.1		52.3 ± 18.8	52.3 ± 5.8	
	Gluconate	33.3 ± 23.8	34.1 ± 1.8		31.8 ± 2.1	34.1 ± 2.5		38.9 ± 30.8	30.7 ± 4.1	
	Malate	23.7 ± 12	21.1 ± 8.9		34.3 ± 11	34.3 ± 5.4		42.1 ± 10.6	44.6 ± 10.8	
	Oxaloacetate	38.3 ± 18.1	34.9 ± 5.1		41.9 ± 14.1	34.6 ± 3.5		19.8 ± 9.2	29.8 ± 6.1	↑^*^
	Pyruvate	29.1 ± 5.2	33.3 ± 0	↑^*^	30 ± 4.5	33.3 ± 0		41 ± 8.9	33.3 ± 0	↓^*^
	Succinate	22.6 ± 12.3	21.2 ± 7.6		31.1 ± 2.8	28.3 ± 4.9		51 ± 8.6	53.7 ± 5.2	
	Threonate	38.3 ± 4.2	35.9 ± 6.4		42.6 ± 5.6	37.6 ± 4.5		18.3 ± 1.8	26.5 ± 5.7	↑^*^
AMINO ACIDS/POLYAMINES	Alanine	26.9 ± 12	33.2 ± 6.6		51.2 ± 6.2	38 ± 6.4	↓^**^	27.2 ± 2.8	28.8 ± 5.2	
	Asparagine	46.7 ± 1	31.3 ± 15.4		37.5 ± 5.7	26.4 ± 13.3		14.6 ± 1.2	22.8 ± 16.4	
	Aspartate	45.1 ± 6.3	47.7 ± 4		36.2 ± 3.1	31.1 ± 4.1	↓^*^	16.8 ± 10.1	22.9 ± 3.8	
	Glutamate	44.4 ± 8.8	47.3 ± 1.1		38.8 ± 5.8	30.9 ± 4.4	↓^*^	16.7 ± 8.9	22.1 ± 4	
	Glutamine	39.8 ± 11.6	21.8 ± 8.4	↓^**^	37.8 ± 4.8	33.7 ± 8.5		19.8 ± 9.9	44.5 ± 14	↑^**^
	Glycine	22.6 ± 11.2	19.2 ± 4		37.1 ± 11.7	32.6 ± 7.7		40.3 ± 10.4	45.6 ± 10.1	
	Isoleucine	26.1 ± 13.2	19.4 ± 4.5		47.7 ± 4.3	31.9 ± 7.7	↓^**^	34.6 ± 6.4	46.6 ± 10.4	↑^*^
	Leucine	27 ± 11.8	20.8 ± 3.8		43.4 ± 13	34 ± 5.6		29.6 ± 10	43.5 ± 8.1	↑^*^
	Lysine	29.3 ± 6.3	18.6 ± 7.6	↓^*^	41.3 ± 12.9	23.2 ± 10.2	↓^*^	29.4 ± 11.4	58.2 ± 16.8	↑^**^
	Methionine	38.4 ± 1.3	33.7 ± 6.1		43.2 ± 4.9	38 ± 4.1	↓^*^	19.5 ± 7.2	28.9 ± 5.7	↑^*^
	Ornithine	35.5 ± 3.9	40.2 ± 25.2		45.9 ± 6.9	29.9 ± 15	↓^*^	18.5 ± 7.5	22 ± 12.9	
	Phenylalanine	25.6 ± 10.1	18.5 ± 7.8		47.4 ± 8.4	26.7 ± 4.1	↓^***^	32.6 ± 10.1	53.2 ± 9.2	↑^**^
	Proline	27.6 ± 8.5	29 ± 2.6		47.1 ± 10.2	37.5 ± 4.9	↓^*^	25.3 ± 9.3	31.6 ± 5.1	
	Serine	28.9 ± 7.7	24.1 ± 2.8		47.7 ± 10.3	37.8 ± 5	↓^*^	23.4 ± 8.7	35.7 ± 6.1	↑^**^
	Threonine	30.6 ± 13	25.9 ± 3.9		45.5 ± 15.9	37.9 ± 5.8		20 ± 4.6	34.1 ± 9.4	↑^*^
	Tryptophan	20.8 ± 8.5	30.9 ± 3.4	↑^**^	30.1 ± 16	33.2 ± 0.4		49.1 ± 23.4	34.6 ± 2.5	
	Tyrosine	25.4 ± 5.7	16.7 ± 10.5		38.9 ± 14.8	19.9 ± 10.9	↓^*^	35.7 ± 14.4	64.2 ± 20.6	↑^*^
	Valine	25.8 ± 12	19.6 ± 3.3		48.3 ± 4.2	32.5 ± 6.3	↓^***^	30.4 ± 11.2	45.8 ± 9.4	↑^*^
	Putrescine	38.2 ± 4	37.8 ± 7.7		40.5 ± 3.5	38 ± 5.3		19.1 ± 5	24.2 ± 3.5	↑^*^
	Spermidine	46.7 ± 4.2	33.4 ± 0.1	↓^***^	33.6 ± 5.7	33.5 ± 0.3		20.7 ± 1.3	32.6 ± 3.4	↑^***^

*Mean values of non-acclimated plants (Non Acc) and acclimated plants (Acc). (n ≥ 6); Asterisks indicate significant changes during acclimation per compartment (ANOVA and Tukey*,

* p < 0.05;

** p < 0.01;

*** *p < 0.001) and arrows (↑; ↓) indicate an increase or decrease*.

Cold-induced differences in the relative distribution were also detected for various amino acids (Table 1). Comparing mean values of relative distribution under control condition, two thirds, i.e., 12 out of 18, of the amino acids were found to have their maximum portion in the cytosolic compartment and only 2 out of 18 had their maximum portion in the vacuolar part of the cell. During cold acclimation, the maximum relative portion of most amino acids was found to be vacuolar. In detail, only tryptophan reached a significantly higher percentage in the chloroplast. A slight increase of the plastidial portion was observed for alanine, aspartate, glutamic acid, ornithine and proline, while all others decreased. In the cytosol, only the relative amount of tryptophan increased while the relative amount of all other amino acids decreased during cold acclimation.

### Cold-induced subcellular reprogramming in context of stress-tolerance

The subcellular fractionation technique was applied to reveal significant differences between the cold-induced metabolic reprogramming in a cold sensitive (Cvi, origin: Cape Verde Islands) and tolerant (Rsch, origin: Russia) accession of *A. thaliana*. In general, and except for melibiose levels in Cvi, sugars were found to accumulate significantly during the acclimation process. Those differences between absolute levels in Cvi and Rsch have been described before for the same experimental setup (Nagler et al., 2015). Here, we now provide the relative distribution of metabolites among cellular compartments (for details see Supplementary Data 4) allowing for the calculation of absolute levels within these compartments (Table 2; Supplementary Data 5). During cold acclimation, soluble carbohydrates as well as sugar alcohols increased significantly in both accessions. Particularly, sucrose, raffinose, glucose and fructose levels increased during the cold exposure and this increase was more pronounced across all compartments in the tolerant accession Rsch. In contrast, levels of melibiose, which is a degradation product of raffinose, significantly decreased only in the cytosol and vacuole of the sensitive accession Cvi. In addition, the observed fold-change of myo-inositol was higher in Cvi than in Rsch for all compartments (Table 2).

**Table 2:** **Comparison of subcellular metabolite levels before and after cold acclimation**.

		**Ratio of Subcellular Absolute Levels: acc vs. non-acc**					
		**CHLOROPLAST**		**CYTOSOL**		**VACUOLE**	
**METABOLITES**		**Cvi**	**Rsch**	**Cvi**	**Rsch**	**Cvi**	**Rsch**
SUGARS/SUGAR ALCOHOLS	Fructose	26.59^***^	81.08^***^	27.75^***^	89.57^***^	35.35^***^	107.36^***^
	Galactinol	1.08	2.21^*^	1.51^*^	3.08^***^	1.08	6.12^*^
	Glucose	17.18^***^	20.68^***^	20.02^***^	21.02^***^	20.58^***^	22.81^***^
	Melibiose	0.96	1.25	0.92^***^	1.05	0.89^**^	1.06
	myo-Inositol	6.40^***^	4.83^***^	9.70^***^	8.49^***^	12.51^***^	9.09^**^
	Raffinose	1.32	87.81^***^	1.22	109.70^***^	2.88^*^	199.74^*^
	Sucrose	3.63^***^	8.77^***^	3.47^***^	13.08^***^	3.54^***^	18.81^**^
	Threitol	0.28^**^	1.28	0.28^**^	1.09	1.53^*^	0.81
ORGANIC ACIDS	2-Oxoglutarate	0.96	0.83	0.87	1.34^*^	0.79	0.81
	Citrate	1.43^*^	6.09^***^	1.13	7.78^***^	0.99	5.88^***^
	Fumarate	6.10^***^	8.13^***^	6.23^***^	5.91^***^	8.86^***^	4.46^***^
	Gluconate	0.94^***^	1.47	0.94^***^	1.42^*^	0.94^***^	0.73
	Malate	3.18^***^	8.54^***^	2.48^***^	9.49^***^	2.63^***^	7.00^***^
	Oxaloacetate	1.47	1.13	1.47	1.04	0.51^*^	1.05
	Pyruvate	0.70^***^	1.31^*^	0.64^***^	1.59^**^	0.64^***^	1.02
	Succinate	0.24^***^	1.74^*^	0.24^***^	2.02^**^	0.37^***^	1.97^***^
	Threonate	0.56^***^	2.09^***^	1.05	2.78^***^	1.28	4.15^**^
AMINO ACIDS/POLYAMINES	Alanine	0.67^**^	1.47^*^	0.86	1.70^**^	1.10	1.43
	Asparagine	0.70	0.80	1.48	1.22	1.84	0.92
	Aspartate	1.64^***^	3.25^***^	1.76^***^	3.79^***^	2.07^***^	2.89^*^
	Glutamate	0.27^***^	1.65^**^	0.32^***^	2.22^***^	0.36^***^	2.72^**^
	Glutamine	0.52^***^	1.74^*^	1.32^*^	2.90^***^	3.44^***^	5.57^**^
	Glycine	1.35	1.48^**^	1.69^***^	2.19^***^	3.66^***^	3.23^***^
	Isoleucine	0.73^*^	1.38	1.29^**^	1.71^**^	1.47^**^	1.87
	Leucine	0.72^*^	1.27	1.16	1.37^*^	1.28	1.31
	Lysine	0.25^**^	0.43^***^	0.47^**^	0.52^**^	2.83^*^	2.54^**^
	Methionine	0.54^*^	0.84	1.24	1.27	1.46	1.00
	Ornithine	0.52	0.81	1.77	1.21	1.84^**^	1.69
	Phenylalanine	0.32^***^	0.85	0.65^**^	1.02	1.48^**^	1.08
	Proline	7.65^***^	41.55^***^	14.86^***^	60.44^***^	13.97^***^	56.76^**^
	Serine	0.56^***^	0.99	1.14	1.48^*^	1.22^*^	1.70
	Threonine	1.54^*^	1.72^*^	1.46^***^	2.40^***^	1.49^**^	2.10^*^
	Tryptophan	0.68	0.91	0.59^*^	0.94	1.41	1.31
	Tyrosine	0.46^*^	0.80	0.51	1.18	2.99^*^	2.53^***^
	Valine	0.74^*^	1.58^**^	1.18	1.84^***^	1.20	1.64^*^
	Putrescine	0.88	1.38^**^	1.88^***^	1.76^***^	3.54^***^	2.22^***^
	Spermidine	0.93^***^	0.82	0.93^***^	0.77^*^	0.93^***^	2.49^**^

*Ratios were built from mean values of cold acclimated and non-cold acclimated samples (acc/non-acc). A detailed summary of all values including information about standard deviation is provided in Supplementary Data 4, 5. Asterisks indicate significant ratios (ANOVA and Tukey*,

* p < 0.05;

** p < 0.01;

*** *p < 0.001)*.

For organic acids the picture of cold-induced changes was more diverse. In Cvi, pyruvate levels decreased significantly in all compartments while they increased in plastids and cytosol of Rsch. Citrate significantly increased in chloroplasts of both accessions, yet this increase was more pronounced in Rsch. Additionally, citrate levels increased significantly in the cytosol and vacuole of Rsch which was not observed for Cvi. 2-oxoglutarate was found to increase significantly only in the cytosol of Rsch. Succinate significantly decreased in Cvi and increased in Rsch across all compartments. Fumarate and malate significantly increased in both accessions and again this increase was found to be more pronounced in Rsch (Table 2).

Accession-specific dynamics of subcellular absolute levels were also detected for several amino acids. The strongest cold-induced alteration was detected for proline levels in Rsch which were increased 60-fold in the cytosol of acclimated plants (Table 2). In Cvi, the strongest proline accumulation was found to occur in the vacuole with a 14-fold increase during cold acclimation. A significantly different picture for both accessions was observed for glutamate which significantly decreased in all compartments of Cvi while it significantly increased in Rsch, pointing to a central role in metabolic reprogramming in the cold tolerant accession.

**Figure 5:**
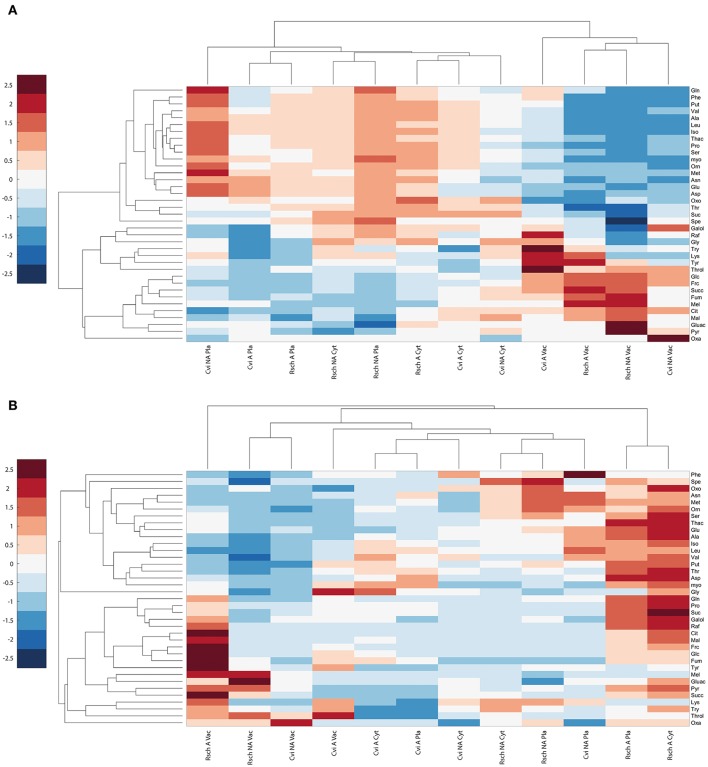
**Cluster analysis of relative and absolute subcellular metabolite distributions in Cvi and Rsch. (A)** Relative distribution of metabolites, **(B)** Absolute levels per compartment. Mean values of relative and absolute levels of non-acclimated (NA) and acclimated (A) samples (*n* ≥ 6) were scaled by zero mean-unit variance (z-scores). Frc, Fructose; Galol, Galactinol; Glc, Glucose; Mel, Melibiose; myo, myo-Inositol; Raf, Raffinose; Suc, Sucrose; Throl, Threitol; Cit, Citrate; Fum, Fumarate; Gluac, Gluconate; Mal, Malate; Oxa, Oxaloacetate; Oxo, 2-Oxoglutarate; Pyr, Pyruvate; Succ, Succinate; Thac, Threonate; Ala, Alanine; Asn, Asparagine; Asp, Aspartate; Glu, Glutamate; Gln, Glutamine; Iso, Isoleucine; Leu, Leucine; Lys, Lysine; Met, Methionine; Orn, Ornithine; Phe, Phenylalanine; Pro, Proline; Ser, Serine; Thr, Threonine; Try, Tryptophan; Tyr, Tyrosine; Val, Valine; Spe, Spermidine; Put, Putrescine; Pla, Plastid; Cyt, Cytosol; Vac, Vacuole.

Hierarchical cluster analysis, which was based on Euclidean distance information of relative subcellular metabolite distribution (Figure 5A), revealed a separation of the vacuolar from the plastidial and cytosolic compartment in both acclimation states. In both genotypes, this separation was due to a high vacuolar percentage of metabolites like threitol, glucose, fructose, fumaric acid, citric acid, and malic acid. *Vice versa*, most amino acids and polyamines showed a higher percental distribution in cytosol and chloroplasts across both analyzed genotypes and conditions (Figure 5A).

Clustering of absolute subcellular metabolite levels revealed a different pattern where acclimated samples of the cold tolerant accession Rsch showed a much stronger increase of metabolite levels across all compartments than Cvi (Figure 5B). Additionally, in chloroplasts and cytosol of Rsch, amino acids, and polyamines showed a much more pronounced accumulation than in Cvi. Also cryoprotective substances like sucrose, raffinose and proline accumulated to higher levels in the cytosolic and plastidial compartments of the tolerant accession Rsch compared to Cvi (Figure 5B; Table 2; Supplementary Data 5 Tables SIII, SIV). Yet, again, and similar to the relative distribution of metabolites, the metabolites glucose, fructose, citric acid, malic acid and fumaric acid increased much stronger in the vacuoles than in other compartments of both genotypes.

## Discussion

Due to the high degree of subcellular compartmentation, the interpretation of metabolome data derived from eukaryotic whole-cell extracts is limited. While protein data can often be interpreted in context of subcellular compartments due to characteristic amino acid sequences and enrichment techniques (Millar and Taylor, 2014), accompanying dynamics of the metabolome cannot be resolved equivalently. Here, we present a method which is capable of resolving the subcellular metabolome in a high-throughput benchtop manner by correlating metabolite abundances with compartment-specific marker enzyme activities. The original methodology of non-aqueous fractionation, which our methodology is based on, has been developed and applied successfully for decades (see e.g., Gerhardt and Heldt, 1984; Stitt et al., 1989; Farre et al., 2008; Klie et al., 2011; Krueger et al., 2011; Nägele and Heyer, 2013; Szecowka et al., 2013; Arrivault et al., 2014), yet several technical difficulties still limit its high-throughput application. In this context, our presented technique allows a benchtop fractionation which replaces technically difficult and time-consuming steps, e.g., ultracentrifugation and density gradient mixing of organic solvents. This is particularly enabled by changing the height of the organic liquid column which the sample has to pass through in each centrifugation step. Classically, to separate the cellular fractions by their specific density, ultracentrifugation is applied using tubes with volumes >10 mL (Stitt et al., 1989). In the presented method, this procedure is replaced by step-wise resuspension of tissue material and centrifugation through a small volume of organic solvent (1 mL) which enables the application of benchtop centrifuges and reduces centrifugation periods significantly. Further, in a typical benchtop centrifuge, more than 20 samples can be processed simultaneously while this is not possible in an ultracentrifuge. Hence, this increases the potential sample throughput considerably and, particularly in combination with microplate readers, pipetting robot platforms and hyphenated chromatography-mass spectrometry methods a comprehensive and statistically robust insight into subcellular metabolome dynamics becomes possible.

A second major advancement is the possibility to adjust gradients of organic solvent in a non-linear manner which is hardly possible by (most) gradient mixings systems. Additionally, testing the quality of mixed colorless organic solvent gradients in an ultracentrifugation tube is not possible which dramatically impacts the quality control of the fractionation method before the time consuming ultracentrifugation step. In our presented procedure such a control of correct solvent density is automatically given as the solution is prepared in the desired density directly before the sample is re-suspended in it.

Third, compared to the original method, which suggests the application of 200–300 mg of dry leaf powder (Gerhardt and Heldt, 1984; Stitt et al., 1989), only a fraction of this is needed in the presented method (e.g., 10–20 mg of dried leaf material). This makes the whole procedure applicable to studies which are limited by material, e.g., *in situ* or “in-field” samples, rare organ or tissue material.

Finally, the step-wise centrifugation of material suspensions allows for additional supporting ultrasonication steps which contribute to a more efficient lysis of cell component fractions being a prerequisite for a successful separation. The efficiency of sonication was already shown for tobacco cells, where sonication cycles of 6 min lead to more than 99% of disrupted cells (Hu and Brown, 1994). This fine-tunes the separation efficiency of cellular compartments and contributes to a higher resolution capability of the method. Particularly with regard to the separation of small organelles, e.g., peroxisomes or mitochondria, which are frequently associated with other subcellular compartments and, hence, difficult to resolve (Arrivault et al., 2014), this might play a crucial role.

When applying the fractionation technique to non-acclimated and acclimated *Arabidopsis* plants of the accession Col-0, marker enzyme activities indicated a reproducible separation of compartments irrespective of the acclimation state (Figure 4). Hence, although leaves might undergo changes in their physical characteristics during cold exposure (Strand et al., 1999), these changes do not seem to affect the quality of separation. However, while the presented method accounts for the subcellular fractionation of homogenized leaf material, it does not resolve the heterogeneity of leaf tissue. Hence, if the portion of epidermal, palisade or mesophyll cells undergoes developmentally or environmentally-induced dynamics, the direct comparison of resolved subcellular metabolite levels will become difficult or, in the worst case, even misleading. A strategy to reduce the resulting ambiguousness of the experimental data could be the application of the presented method to isolated protoplasts or a combination of the presented method with single-cell methods, e.g., imaging methods (Wuyts et al., 2010) or laser ablation electrospray ionization (LAESI) mass spectrometry (MS) (see e.g., Li et al., 2015).

Applying the suggested calculation algorithm as described (see Figure 3), several significant cold-induced shifts of metabolite distribution were detected. A dominating effect was the shift of relative amounts of sugars, sugar alcohols and amino acids from the cytosol into the vacuole (see Table 1). Hence, these findings provide evidence for a central role of the cytosol-vacuole interaction during cold-acclimation in Col-0. The importance of the vacuolar compartment in cold-induced metabolic reprogramming has also been indicated in a previous tonoplast proteome study which could identify several membrane proteins which were altered in their abundance during cold acclimation (Schulze et al., 2012). Based on their observations, Schulze and colleagues concluded that cold-induced vacuolar solute accumulation occurs due to increased acidification, and transport activity across the tonoplast was suggested to be modulated by protein amounts as well as phosphorylation states. Another study provided evidence for a central role of proton-coupled vacuolar glucose transport in the development of freezing tolerance (Klemens et al., 2014). While this substantiates our indications for a significant role of vacuolar sugar metabolism during cold acclimation, the role of vacuolar reprogramming of amino acid metabolism seems less clear. The relative distribution of amino acids in Col-0 was shifted toward the vacuole during cold acclimation whereas the plastid-cytosol interaction was less affected (see Table 1). Due to the fact that the chloroplast plays a central role in the regulation of various amino acid-related pathways, e.g., the biosynthetic pathways of glutamate, glutamine, branched chain and aromatic amino acids (Kleffmann et al., 2004; Maeda and Dudareva, 2012), a possible explanation for our observation would be that the vacuole buffers subcellular re-arrangements against environmental fluctuations. This could contribute to the stabilization of the plastidial amino acid metabolism playing a crucial role in the whole plant C/N homeostasis.

To reveal whether this hypothesis of vacuolar buffering of amino acid metabolism might also be evident for other natural accessions of *Arabidopsis*, we further analyzed the cold-induced subcellular metabolome dynamics in the cold-sensitive and tolerant accessions Cvi and Rsch, respectively. Indeed, we observed a more significant shift of amino acids between plastid, cytosol and vacuole in the sensitive Cvi accession, while in Rsch only a few metabolites were affected in their relative distribution during cold acclimation. Hence, to summarize our findings about relative shifts of metabolite levels during cold acclimation with respect to the freezing tolerance of the considered accessions (Cvi < Col < Rsch, see e.g., Hannah et al., 2006) we suggest that the intensity of significant subcellular re-arrangement of primary metabolism is negatively correlated with freezing tolerance. In contrast, the absolute amount of substance of many sugars, amino acids and organic acids has been shown to positively correlate with freezing tolerance, not only on a cellular (Klotke et al., 2004; Nagler et al., 2015) but also on a subcellular level (Nägele and Heyer, 2013). These significant differences between absolute subcellular metabolite levels became also clear in the present study (Figure 5B; Table 2; Supplementary Data 5 Tables SIII, SIV). While the molecular reasons for these differences remain elusive it is tempting to speculate that the regulation of the energy homeostasis plays a crucial role and might differ between Cvi and Rsch. While the cold sensitive accession Cvi seems to be characterized by a strong cold-induced shuffling of metabolites between subcellular compartments, the cold- and freezing-tolerant accession Rsch might coordinate its metabolism rather in direction of biosynthesis than intracellular transport of cryoprotective substances. Of course, it is not surprising that energy balance plays a central role in cold acclimation and stress tolerance as it has already been nicely summarized almost two decades ago (Huner et al., 1998). However, recent advances in understanding a plant's energy homeostasis and its reprogramming due to environmental fluctuation and stress conditions have revealed a highly complex regulatory and biochemical network (for an overview see e.g., Tome et al., 2014; Provart et al., 2016). These metabolic and regulatory networks comprise various cellular organelles and their interaction via signaling cascades and shuttles. Hence, it can be expected that a comprehensive, reliable and realistic picture of eukaryotic metabolism and its control can only arise if the subcellular compartmentation of metabolism is considered in experimental studies. Our presented methodology for cellular fractionation aims at supporting these biochemical and physiological studies. Conclusively, the output gained from subcellular studies combining relative with absolute metabolite levels can profoundly enhance the output of metabolomics studies and helps to unravel cellular players and regulatory strategies which cannot be inferred from whole-cell approaches.

## Experimental procedures

### Plant cultivation and sampling

Plants of the *A. thaliana* accessions Col-0, Cvi and Rsch were cultivated in a growth chamber under controlled conditions. The substrate was composed of Einheitserde ED63 and perlite. Plants were watered daily and fertilized once with NPK fertilization solution (WUX-AL Super; MANNA°-Dünger, Ammerbuch). Light intensity was set to 75 μmol m^−2^ s^−1^ in an 8/16 h day/night cycle and relative humidity to 70 % with a temperature of 22°/16°C. Following 28 days after of sowing light intensity was increased to 125 μmol m^−2^ s^−1^ in a 16/8 h day/night cycle. Bolting stage was reached 43 days after sowing and samples of non-acclimated (non-acc, NA) plants were collected from the three accessions at midday, i.e., after 8 h in the light. One biological sample consisted of 3 leaf rosettes which were immediately quenched in liquid nitrogen and stored until further use at −80°C. Non-sampled plants were transferred to 5°C for acclimation (acc, A) with conditions of light intensity, cycle and humidity as before. After 7 days at 5°C, leaf rosettes were sampled as described for non-acclimated plants.

### Measurements of marker enzyme activities

One fraction aliquot was used for photometric (Multiscan Spectrum, Thermo Scientific) determination of three marker enzymes. Samples were extracted in 1 mL extraction buffer consisting of 50 mM Tris-HCl pH 7.3, 5 mM MgCl_2_, and 1 mM DTT. The marker for the plastidial compartment was alkaline pyrophosphatase as described previously (Jelitto et al., 1992) with modifications of amounts to down-scale the assay to a total volume of 200 μL to make it suitable for photometric measurement in a 96 well-plate (Crystal Clear, Greiner Bio One). The cytosolic marker was uridinediphosphate glucose pyrophosphorylase (UGPase) as described in Zrenner et al. (1993) with modifications regarding recalculations of amounts in a 96 well-plate (μClear, Greiner Bio One). Acid phosphatase was used as a marker for the vacuolar compartment as described in Boller and Kende (1979), again adapted for a 96 well plate (Crystal Clear, Greiner Bio One).

### GC-MS analysis of subcellular metabolite levels

Metabolites were extracted from subfractions according to Weckwerth et al. (2004) with slight modifications. 1 mL of −20°C cold methanol/chloroform/H_2_O (2.5/1/0.5 v/v/v) mixture was added to each sample fraction. Samples were vortexed, incubated on ice for 10 min and centrifuged for 4 min at 4°C and 20.000 g. 300 μL H_2_O were added to the supernatant, followed by brief vortexing and 2 min of centrifugation. Following the separation of the polar methanol/water phase from the unpolar chloroform phase, samples were dried in a vacuum concentrator (LaboGene™, Denmark) for derivatization. The dried pellets were resolved at 30°C for 90 min in 20 μL of a 40 mg mL^−1^ methoxyamine hydrochloride in pyridine solution. Afterwards, 80 μL of N-methyl-N-trimethylsilyltrifluoroacetamid (MSTFA), spiked with 30 μL mL^−1^ of a mix of even-numbered alkanes (C_10_-C_40_), were added, and the samples were incubated for 30 min at 37°C under constant shaking, followed by 2 min of centrifugation. The supernatant was transferred into a glass vial for measurement. GC-MS measurements were performed on an Agilent 6890 gas chromatograph coupled to a LECO Pegasus® 4D GCxGC-TOF mass spectrometer (LECO® Corporation, Michigan, USA). For GC analysis, the initial oven temperature was set to 70°C for 1 min, followed by a 9°C min^−1^ ramp with 330°C end temperature which was set constant for 8 min. In the MS method, the data acquisition rate was set to 20 spectra sec^−1^ at a detector voltage of 1550 V. The acquisition delay was set to 5.5 min and the detected mass range was set from 40 to 600 m/z. Raw data were processed with the LECO Chroma-TOF® software (LECO® Corporation, Michigan, USA). Measurements of non-acclimated samples were performed in a splitless measurement mode (1:1) while cold acclimated samples were measured in split mode (1:2). Relative distribution of metabolites was calculated based on the quantified area in the chromatograms.

### Data analysis and statistics

Data evaluation was performed in Microsoft Excel (http://www.microsoft.com). Outlier identification and hierarchical cluster analysis was performed within the numerical software Matlab (www.mathworks.com). Analysis of Variance (ANOVA) and Tukey *post-hoc* test were done in the R software environment (The R Project for Statistical Computing; http://www.r-project.org/).

## Author contributions

LF performed experiments, data evaluation and wrote the paper. WW performed data evaluation and wrote the paper. TN conceived the study, performed data evaluation and wrote the paper.

## Funding

This work was supported by the Austrian Science Fund (FWF Project number I2071).

### Conflict of interest statement

The authors declare that the research was conducted in the absence of any commercial or financial relationships that could be construed as a potential conflict of interest.

## Supplementary material

Supplementary Image 1 **Suspension of lyophilized leaf material. (A,B)** Sample before and **(C,D)** after sonication. **(A,C)** Leaf material suspension; **(B,D)** light microscopy pictures reveal the effect of sonication on leaf cells.

Supplementary Image 2 **Results of subcellular fractionation steps. (A–C)** Fractions of different density after step 11 (see Figure 2). **(D–F)** Fractions after step 14 before drying (see Figure 2).

Supplementary Data 1 **Examples for density gradient modifications**.

Supplementary Data 2 **Description of calculation algorithm**.

Supplementary Data 3 **Fill-in form for measurements**.

Supplementary Data 4 **Relative compartmental distribution of metabolites in Cvi and Rsch**.

Supplementary Data 5 **Absolute compartmental distribution of metabolites in Cvi and Rsch**.
