# Supplementary figures and images for: A Benchtop Fractionation Procedure for Subcellular Analysis of the Plant Metabolome
Source: Front Plant Sci. 2016 Dec 22;7:1912. doi: 10.3389/fpls.2016.01912 (PMC5177628; doi:10.3389/fpls.2016.01912)

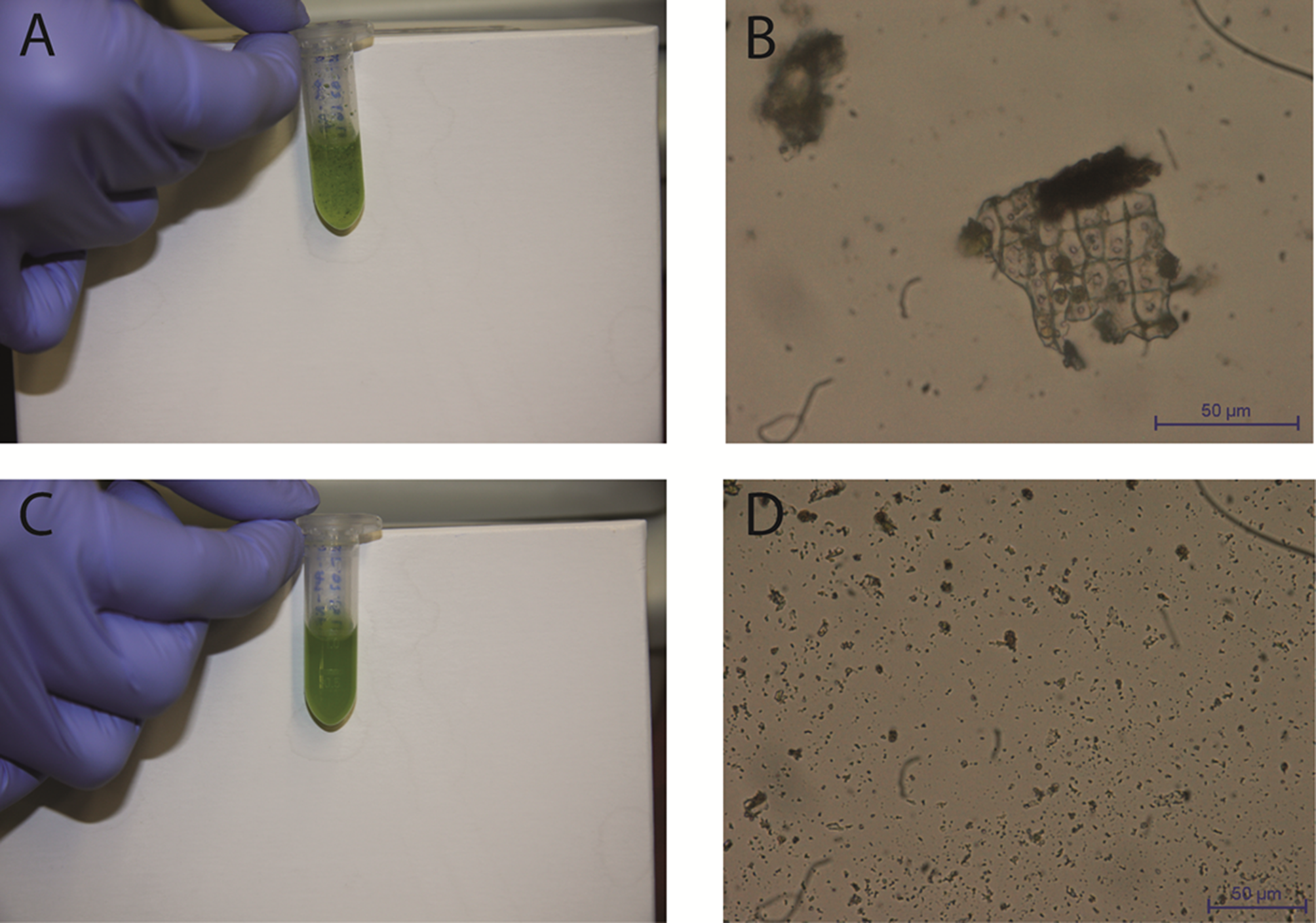

Supplement: Supplementary Image 1 — Suspension of lyophilized leaf material. (A,B) Sample before and (C,D) after sonication. (A,C) Leaf material suspension; (B,D) light microscopy pictures reveal the effect of sonication on leaf cells. [file Image1.TIF]

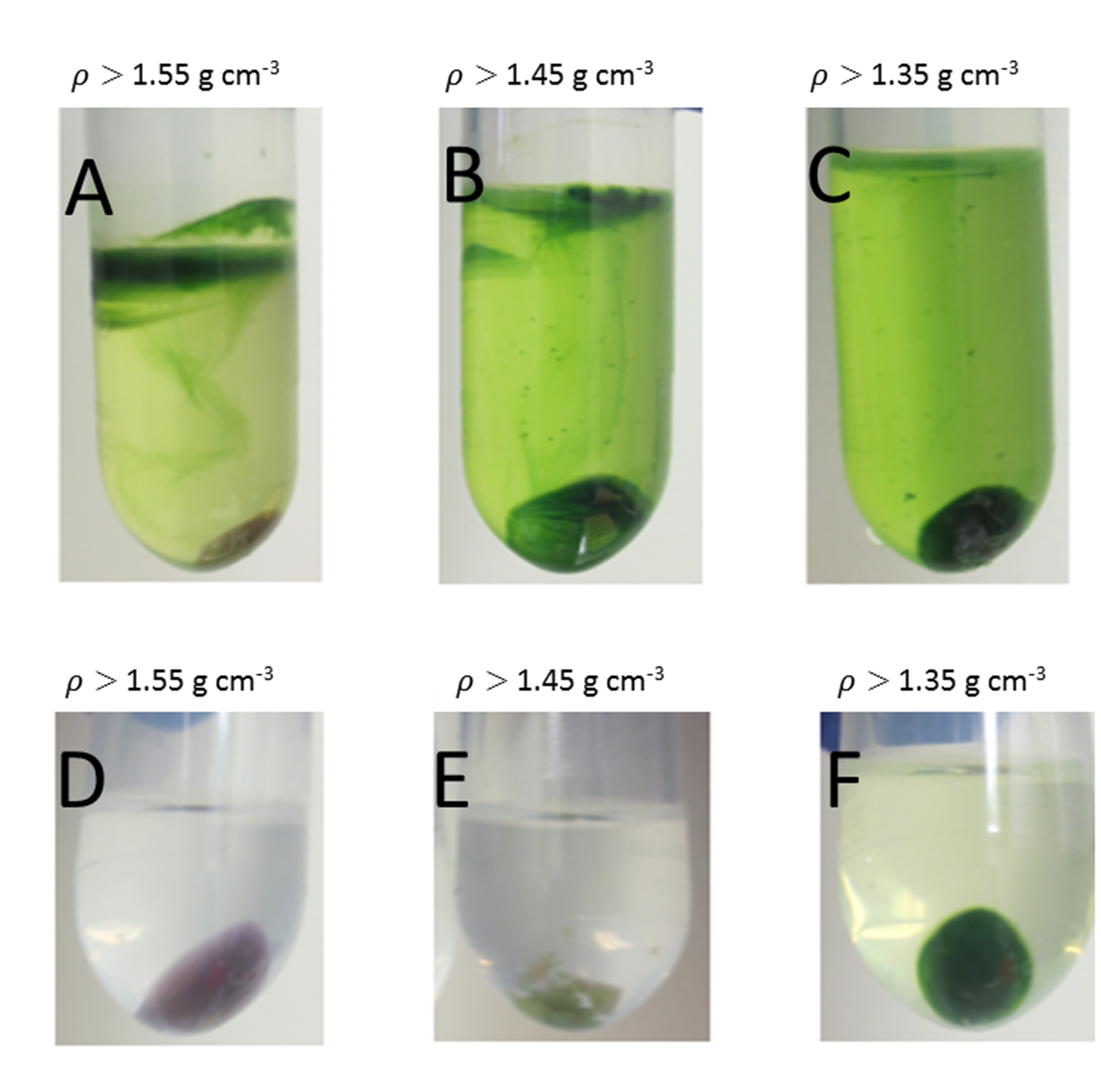

Supplement: Supplementary Image 2 — Results of subcellular fractionation steps. (A–C) Fractions of different density after step 11 (see Figure 2). (D–F) Fractions after step 14 before drying (see Figure 2). [file Image2.TIF]
