# Supplementary material for: A Benchtop Fractionation Procedure for Subcellular Analysis of the Plant Metabolome
Source: Front Plant Sci. 2016 Dec 22;7:1912. doi: 10.3389/fpls.2016.01912 (PMC5177628; doi:10.3389/fpls.2016.01912)
Supplement: Supplementary Data 1 — Examples for density gradient modifications. [file DataSheet1.PDF]

Supplementary Data 1. Examples for Density Gradient Modifications

Example Gradients

a

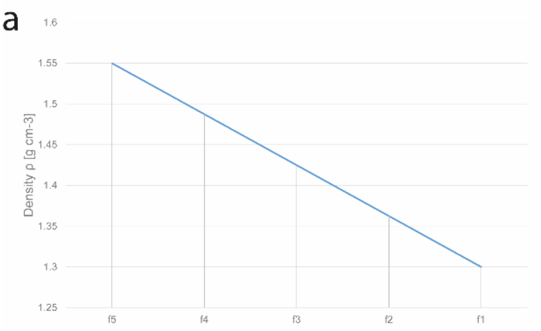

a) Linear Gradient

fraction	*f5*	*f4*	*f3*	*f2*	*f1*
density	1.55	1.49	1.43	1.36	1.3
+7H	(+57.5 µl)	+78.3 µl	+90.9 µl	+126.3 µl	+130.1 µl

b

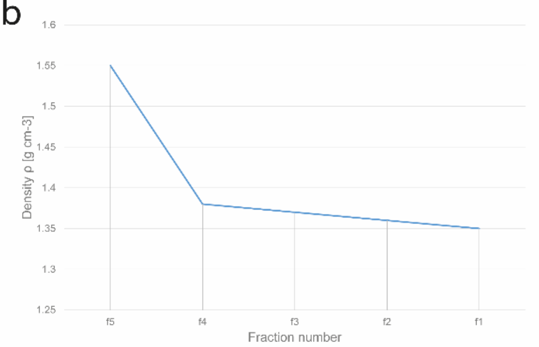

b) Fast drop

fraction	*f5*	*f4*	*f3*	*f2*	*f1*
density	1.55	1.38	1.37	1.36	1.35
+7H	(+57.5 µl)	+256.8 µl	+19.0 µl	+19.6 µl	+20.2 µl

c

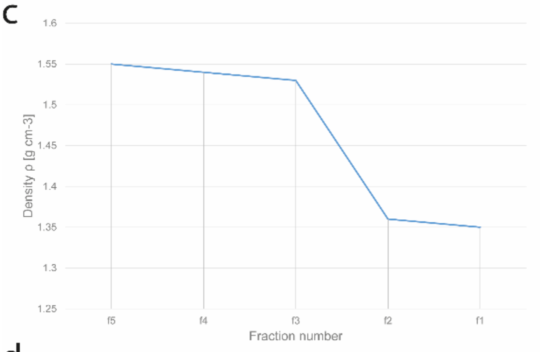

c) Middle Drop

fraction	*f5*	*f4*	*f3*	*f2*	*f1*
density	1.55	1.54	1.53	1.36	1.35
+7H	(+57.5 µl)	+12.3 µl	+12.6 µl	+270.6 µl	+20.2 µl

d

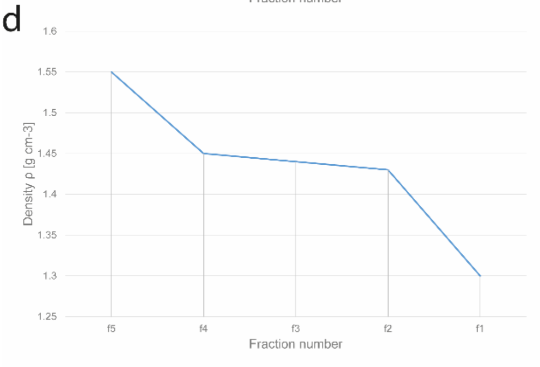

d) Plateau Gradient

fraction	*f5*	*f4*	*f3*	*f2*	*f1*
density	1.55	1.45	1.44	1.43	1.3
+7H	(+57.5 µl)	+137.3 µl	+15.7 µl	+16.1 µl	+257.2 µl
