# Supplementary material for: A Benchtop Fractionation Procedure for Subcellular Analysis of the Plant Metabolome
Source: Front Plant Sci. 2016 Dec 22;7:1912. doi: 10.3389/fpls.2016.01912 (PMC5177628; doi:10.3389/fpls.2016.01912)
Supplement: Supplementary Data 2 — Description of calculation algorithm. [file DataSheet2.PDF]

Supplement 2 - Description of Calculation Algorithm

In total, f fractions ($i, j = \{1, \dots, f\} \mid i < j$) and m metabolites ($x = \{1, \dots, m\}$) resulted from the experimental fractionation procedure. Briefly, the difference of the slope from one fraction to the others was calculated, resulting in binomial coefficient G= $\binom{f}{2}$ possibilities (2-element subset of a f-element set, $G = \{\{i, j\} \mid i < j \; und \; i, j \in \{1, \dots, f\}\}$).

Step 1 – Relative Distribution:

The overall relative distribution of a compartment was determined by enzymatic measurements of marker enzymes in every fraction of a gradient. For further analysis, values of a single fraction (relative distribution [%] in fraction i from plastidial ${}_{Pla}EA_i$, cytosolic ${}_{Cyt}EA_i$ and vacuolar ${}_{Vac}EA_i$ compartment (EA Enzymatic Activity)). For every metabolite, we calculated the relative distribution from GC-MS data over a gradient using quantified compound areas and used values from a single fraction (here described as ${}^{Met_x}GC_i$ relative entry of metabolite 'x' in fraction number i [%]).

Step 2a – Differences among Fractions for Metabolites:

Next, the difference of every two-element subset of the f-element set for every metabolite was built (here inserted relative distribution from GC-MS determined area among all f fractions). Hence, this resulted in $\binom{f}{2} * m$ differences (m= Number of all measured metabolites).

Fraction	*Term*	*Metabolite*$_1$	$\cdots$	*Metabolite*$_x$	$\cdots$	*Metabolite*$_m$
$(1 \rightarrow 2)$	${}^{Met}\Delta^{GC}_{1,2}$	${}^{Met_1}\Delta^{GC}_{1,2} = {}^{Met_1}GC_1 - {}^{Met_1}GC_2$	$\cdots$	${}^{Met_x}\Delta^{GC}_{1,2} = {}^{Met_x}GC_1 - {}^{Met_x}GC_2$	$\cdots$	${}^{Met_m}\Delta^{GC}_{1,2} = {}^{Met_m}GC_1 - {}^{Met_m}GC_2$
$\vdots$	$\vdots$	$\vdots$				$\vdots$
$(1 \rightarrow f)$	${}^{Met}\Delta^{GC}_{1,f}$	${}^{Met_1}\Delta^{GC}_{1,f} = {}^{Met_1}GC_1 - {}^{Met_1}GC_f$				${}^{Met_m}\Delta^{GC}_{1,f} = {}^{Met_m}GC_1 - {}^{Met_m}GC_f$
$(2 \rightarrow 3)$	${}^{Met}\Delta^{GC}_{2,3}$	${}^{Met_1}\Delta^{GC}_{2,3} = {}^{Met_1}GC_2 - {}^{Met_1}GC_3$		$\ddots$		${}^{Met_m}\Delta^{GC}_{2,3} = {}^{Met_m}GC_2 - {}^{Met_m}GC_3$
$\vdots$	$\vdots$	$\vdots$				$\vdots$
$(2 \rightarrow f)$	${}^{Met}\Delta^{GC}_{2,f}$	${}^{Met_1}\Delta^{GC}_{2,f} = {}^{Met_1}GC_2 - {}^{Met_1}GC_f$				${}^{Met_m}\Delta^{GC}_{2,f} = {}^{Met_m}GC_2 - {}^{Met_m}GC_f$
$(i \rightarrow j)$	${}^{Met}\Delta^{GC}_{i,j}$	${}^{Met_1}\Delta^{GC}_{i,j} = {}^{Met_1}GC_i - {}^{Met_1}GC_j$		${}^{Met_x}\Delta^{GC}_{i,j} = {}^{Met_x}GC_i - {}^{Met_x}GC_j$		${}^{Met_m}\Delta^{GC}_{i,j} = {}^{Met_m}GC_i - {}^{Met_m}GC_j$
$\vdots$	$\vdots$	$\vdots$				$\vdots$
$(i \rightarrow f)$	${}^{Met}\Delta^{GC}_{i,f}$	${}^{Met_1}\Delta^{GC}_{i,f} = {}^{Met_1}GC_i - {}^{Met_1}GC_f$		$\ddots$		${}^{Met_m}\Delta^{GC}_{i,f} = {}^{Met_m}GC_i - {}^{Met_m}GC_f$
$\vdots$	$\vdots$	$\vdots$				$\vdots$
$(f-1 \rightarrow f)$	${}^{Met}\Delta^{GC}_{f-1,f}$	${}^{Met_1}\Delta^{GC}_{f-1,f} = {}^{Met_1}GC_{f-1} - {}^{Met_1}GC_f$	$\cdots$	${}^{Met_x}\Delta^{GC}_{f-1,f\;f-1,f} = {}^{Met_x}GC_{f-1} - {}^{Met_x}GC_f$	$\cdots$	${}^{Met_m}\Delta^{GC}_{f-1,f\;f-1,f} = {}^{Met_m}GC_{f-1} - {}^{Met_m}GC_f$

The grey box there is an example of the difference from metabolites between the relative distributions of fraction i to fraction j ('two fixed fractions'), the turquoise box is needed in Step 3.

Step 2b – Differences among Fractions for Enzymatic Activities:

Same procedure as in Step 2a, but here the data are the relative distribution of enzyme activities measurements resulting in $\binom{f}{2} * 3$ deltas (3 for three compartments).

Fraction	*Term*	*Plastid*	*Cytosol*	*Vacuole*
$(1 \rightarrow 2)$	${}_{Comp}\Delta_{1,2}^{EA}$	${}_{Pla}\Delta_{1,2}^{EA} = {}_{Pla}EA_1 - {}_{Pla}EA_2$	${}_{Cyt}\Delta_{1,2}^{EA} = {}_{Cyt}EA_1 - {}_{Cyt}EA_2$	${}_{Vac}\Delta_{1,2}^{EA} = {}_{Vac}EA_1 - {}_{Vac}EA_2$
$\vdots$	$\vdots$	$\vdots$	$\vdots$	$\vdots$
$(1 \rightarrow f)$	${}_{Comp}\Delta_{1,f}^{EA}$	${}_{Pla}\Delta_{1,f}^{EA} = {}_{Pla}EA_1 - {}_{Pla}EA_f$	${}_{Cyt}\Delta_{1,f}^{EA} = {}_{Cyt}EA_1 - {}_{Cyt}EA_f$	${}_{Vac}\Delta_{1,f}^{EA} = {}_{Vac}EA_1 - {}_{Vac}EA_f$
$(2 \rightarrow 3)$	${}_{Comp}\Delta_{2,3}^{EA}$	${}_{Pla}\Delta_{2,3}^{EA} = {}_{Pla}EA_2 - {}_{Pla}EA_3$	${}_{Cyt}\Delta_{2,3}^{EA} = {}_{Cyt}EA_2 - {}_{Cyt}EA_3$	${}_{Vac}\Delta_{2,3}^{EA} = {}_{Vac}EA_2 - {}_{Vac}EA_3$
$\vdots$	$\vdots$	$\vdots$	$\vdots$	$\vdots$
$(2 \rightarrow f)$	${}_{Comp}\Delta_{2,f}^{EA}$	${}_{Pla}\Delta_{2,f}^{EA} = {}_{Pla}EA_2 - {}_{Pla}EA_f$	${}_{Cyt}\Delta_{2,f}^{EA} = {}_{Cyt}EA_2 - {}_{Cyt}EA_f$	${}_{Vac}\Delta_{2,f}^{EA} = {}_{Vac}EA_2 - {}_{Vac}EA_f$
$\vdots$	$\vdots$	$\vdots$	$\vdots$	$\vdots$
$(i \rightarrow j)$	${}_{Comp}\Delta_{i,j}^{EA}$	${}_{Pla}\Delta_{i,j}^{EA} = {}_{Pla}EA_i - {}_{Pla}EA_j$	${}_{Cyt}\Delta_{i,j}^{EA} = {}_{Cyt}EA_i - {}_{Cyt}EA_j$	${}_{Vac}\Delta_{i,j}^{EA} = {}_{Vac}EA_i - {}_{Vac}EA_j$
$\vdots$	$\vdots$	$\vdots$	$\vdots$	$\vdots$
$(i \rightarrow f)$	${}_{Comp}\Delta_{i,f}^{EA}$	${}_{Pla}\Delta_{i,f}^{EA} = {}_{Pla}EA_i - {}_{Pla}EA_f$	${}_{Cyt}\Delta_{i,f}^{EA} = {}_{Cyt}EA_i - {}_{Cyt}EA_f$	${}_{Vac}\Delta_{i,f}^{EA} = {}_{Vac}EA_i - {}_{Vac}EA_f$
$\vdots$	$\vdots$	$\vdots$	$\vdots$	$\vdots$
$(f-1 \rightarrow f)$	${}_{Comp}\Delta_{f-1,f}^{EA}$	${}_{Pla}\Delta_{f-1,f}^{EA} = {}_{Pla}EA_{f-1} - {}_{Pla}EA_f$	${}_{Cyt}\Delta_{f-1,f}^{EA} = {}_{Cyt}EA_{f-1} - {}_{Cyt}EA_f$	${}_{Vac}\Delta_{f-1,f}^{EA} = {}_{Vac}EA_{f-1} - {}_{Vac}EA_f$

In the grey box there are the deltas marked for the compartments of fraction i to fraction j.

Step 3 – Determine Minimum:

The absolute difference of a metabolite to every compartments within two fixed fractions (i,j) is formed in the term ${}^{Met_x}_{Comp}\Psi_{i,j}$ to see correlations between the slopes.

$$ {}^{Met_x}_{Pla}\Psi_{i,j} = \left| {}^{Met_x}\Delta^{GC}_{i,j} - {}_{Pla}\Delta^{EA}_{i,j} \right| \quad , \quad {}^{Met_x}_{Cyt}\Psi_{i,j} = \left| {}^{Met_x}\Delta^{GC}_{i,j} - {}_{Cyt}\Delta^{EA}_{i,j} \right| \quad , \quad {}^{Met_x}_{Vac}\Psi_{i,j} = \left| {}^{Met_x}\Delta^{GC}_{i,j} - {}_{Vac}\Delta^{EA}_{i,j} \right| $$

The minimum for those two fixed fractions (i,j) among the compartments for the metabolite x is determined in the term of ${}^{Met_x}_{Comp}Min_{i,j}$.

$$ {}^{Met_x}_{Comp}Min_{i,j} = Min\left\{ {}^{Met_x}_{Pla}\Psi_{i,j} \quad , \quad {}^{Met_x}_{Cyt}\Psi_{i,j} \quad , \quad {}^{Met_x}_{Vac}\Psi_{i,j} \right\} $$

Resulting in $\binom{f}{2} * m$ minima:

$Fraction$	$Min({}^{Met_1}_{Comp}\Psi)_{Fraction}$		$Min({}^{Met_x}_{Comp}\Psi)_{Fraction}$		$Min({}^{Met_m}_{Comp}\Psi)_{Fraction}$
$(1 \rightarrow 2)$	${}^{Met_1}_{Comp}Min_{1,2}$	$\cdots$	${}^{Met_x}_{Comp}Min_{1,2}$	$\cdots$	${}^{Met_m}_{Comp}Min_{1,2}$
	$\vdots$	$\ddots$			$\vdots$
$(i \rightarrow j)$	${}^{Met_1}_{Comp}Min_{i,j}$		${}^{Met_x}_{Comp}Min_{i,j}$		${}^{Met_m}_{Comp}Min_{i,j}$
	$\vdots$			$\ddots$	$\vdots$
$(f-1 \rightarrow f)$	${}^{Met_1}_{Comp}Min_{f-1,f}$	$\cdots$	${}^{Met_x}_{Comp}Min_{f-1,f}$	$\cdots$	${}^{Met_m}_{Comp}Min_{f-1,f}$

The blue box here indicate the determined minimum for metabolite x in the fractions i and j.

Step 4 – Include upper and lower bounds:

The inclusion of technical errors in the calculation process was performed via error bounds (5 %, 7.5 % and 10 %). The difference between the Ψ candidates and the determined minimum was built and compared to these error bound:

$$ {}^{Met_x}_{Comp}\Psi_{i,j} - {}^{Met_x}_{Comp}Min_{i,j} < \%_{\text{bound}} $$

If the inequality was true, the corresponding compartment was counted as ‘compartment hit’ for every error bound calculation (see step 6).

Step 5 – Consideration of Special Cases:

As written in the main text, a special case was given, when GC-MS detection determined a metabolite to be present in only one fraction – as a result no differences in step 2a and 3 can be determined - therefore for this fraction the compartment hit showing the highest relative marker enzyme activity was chosen as a hit. The creation of a relative distribution therefore is redundant, and Step 6 can be skipped. To include the reliability of the enzymatic measurements, the security intervals are included (e.g. a hit for one compartment resulted in 85 % for this compartment and in 7.5 % for both the others).

Step 6 – Summation and Relative Distribution of Compartment Hits:

For every metabolite a data matrix of compartment hits was registered, as exemplarily shown here:

$Metabolite_x$	$< 5\%_{bound}$	$< 7.5\%_{bound}$	$< 10\%_{bound}$
$Compartment\ Hit(1 \rightarrow 2)$	*Cyt*	*Cyt*<br>*Vac*	*Cyt*<br>*Vac*
$\vdots$			
$Compartment\ Hit(i \rightarrow j)$	*Cyt*<br>*Vac*	*Cyt*<br>*Vac*	*Pla*<br>*Cyt*<br>*Vac*
$\vdots$			
$Compartment\ Hit(f-1 \rightarrow f)$	*Vac*	*Vac*	*Vac*
$\sum('Pla')$	3	3	4
$\sum('Cyt')$	5	5	6
$\sum('Vac')$	7	8	8

The specific hits for compartments were summed up and afterwards the relative distribution for the specific metabolite in the compartments was determined for every security interval. The arithmetic mean and standard deviation of all three security interval distributions for every compartment were built and the final term ${}^{Met_x}_{Comp}\%$ was introduced.

Step 7 – Calculation of Absolute subcellular Levels

Absolute determined metabolite concentrations from unfractionated extracts were inserted in the relative distribution of metabolites combined with the compartments (${}^{Met_x}_{Comp}\%$) and resulted in the term ${}^{Met_x}_{Comp}Abs$, the absolute concentrations were also inserted in the standard deviation of Step 6.
