# Supplementary material for: A Benchtop Fractionation Procedure for Subcellular Analysis of the Plant Metabolome
Source: Front Plant Sci. 2016 Dec 22;7:1912. doi: 10.3389/fpls.2016.01912 (PMC5177628; doi:10.3389/fpls.2016.01912)
Supplement: Supplementary Data 4 — Relative compartmental distribution of metabolites in Cvi and Rsch. [file DataSheet4.PDF]

Supplement 4 - Relative Distribution of metabolites in Cvi and Rsch per compartment

Table SI: Relative Distribution (Mean Values ± Standard Deviation) of Cvi before and after cold acclimation. Asterisks indicate significant changes during acclimation per compartment (ANOVA & Tukey, * p<0.05; ** p<0.01; *** p<0.001) arrows (↑; ↓) indicate direction during acclimation.

Cvi [%]	Chloroplast			Cytosol			Vacuole		
**Metabolite**	**Non-Acc**	**Acc**	**p**	**Non-Acc**	**Acc**	**p**	**Non-Acc**	**Acc**	**p**
**Fructose**	23.5 ± 7.1	20.1 ± 7.4		33.5 ± 2.3	29.8 ± 4.8		44.2 ± 10.2	50.1 ± 7.9	
**Galactinol**	23.5 ± 10.8	20.5 ± 4.9		33.7 ± 6.2	41 ± 1.9	↑*	47.1 ± 21.5	41.1 ± 5.8	
**Glucose**	22.6 ± 3.6	19.8 ± 8.2		29.3 ± 6.4	29.9 ± 6.2		48 ± 7.9	50.3 ± 8.2	
**Melibiose**	31.9 ± 3.2	33.3 ± 0		33.3 ± 0	33.3 ± 0		34.5 ± 2.6	33.3 ± 0	
**myo-Inositol**	47.4 ± 5.1	37.3 ± 9.2	↓*	33.8 ± 2.1	40.3 ± 2.3	↑**	17.3 ± 0.9	26.6 ± 3.4	↑**
**Raffinose**	22.2 ± 13.7	18.5 ± 9.3		34.4 ± 2.1	26.5 ± 19.2		30.4 ± 14.7	55.1 ± 25.7	
**Sucrose**	29.9 ± 6.6	30.6 ± 7.7		42.1 ± 3.1	41.3 ± 7.8		28 ± 4.1	28 ± 4.3	
**Threitol**	24.7 ± 13.3	7.5 ± 0	↓*	24.7 ± 13.3	7.5 ± 0	↓*	50.6 ± 26.7	85 ± 0	↑*
**2-Oxoglutarate**	33.9 ± 7.5	36.3 ± 6.7		38.6 ± 5.6	37.2 ± 5.2		27.5 ± 7.4	24.2 ± 3.5	
**Citrate**	17.2 ± 1.4	22.7 ± 6		38.4 ± 7.2	40.1 ± 4.5		43.2 ± 4.9	39.7 ± 2.6	
**Fumarate**	23.5 ± 3.5	19.7 ± 6.8		41.4 ± 3	35.4 ± 5.8	↓*	34.6 ± 0.7	42.1 ± 5.3	↑*
**Gluconate**	33.3 ± 0	33.3 ± 0		33.3 ± 0	33.3 ± 0		33.3 ± 0	33.3 ± 0	
**Malate**	21.8 ± 3.9	26.3 ± 8.1		42.1 ± 0.8	39.5 ± 2.7		34.2 ± 2.1	34.2 ± 6.4	
**Oxaloacetate**	20.4 ± 14.1	33.3 ± 0	↑*	20.4 ± 14.1	33.3 ± 0	↑*	59.2 ± 28.3	33.3 ± 0	↓*
**Pyruvate**	31.9 ± 4.2	33.3 ± 0		34.8 ± 2.2	33.3 ± 0		35.1 ± 2.6	33.3 ± 0	
**Succinate**	26.7 ± 2.6	23.1 ± 9		39.9 ± 1.7	34.4 ± 5.7	↓*	31.7 ± 5.3	42.5 ± 10.2	↑*
**Threonate**	54.8 ± 1.5	35.1 ± 4.6	↓***	31.2 ± 5.3	37.7 ± 5.8		16.1 ± 3.3	23.8 ± 5	↑*
**Alanine**	43.2 ± 8.3	35.8 ± 8.6		35.8 ± 7.5	37.9 ± 8.2		19.5 ± 1.2	26.2 ± 2.8	↑***
**Asparagine**	49.2 ± 35.1	44.1 ± 28.8		15.5 ± 11.9	29.1 ± 17.7		11.4 ± 5.5	26.7 ± 32.6	
**Aspartate**	54.1 ± 3.2	49.9 ± 5.1		29.3 ± 5.4	28.8 ± 3.1		18.4 ± 4.6	21.3 ± 4.7	
**Glutamate**	56 ± 1.7	48.5 ± 6.9	↓*	28.6 ± 5.7	29.7 ± 2.2		18.8 ± 1.5	21.8 ± 6	
**Glutamine**	57.7 ± 4	24 ± 7.1	↓***	29.4 ± 6.5	31.2 ± 4.4		15.1 ± 3.1	41.7 ± 4.4	↑***
**Glycine**	33.9 ± 5.7	21.7 ± 5.8	↓**	42.3 ± 3.8	33.9 ± 3.7	↓**	23.9 ± 6.4	41.5 ± 8.3	↑**
**Isoleucine**	49.2 ± 11	37.2 ± 6.5	↓*	30.2 ± 5.2	40.3 ± 1.8	↑**	17.6 ± 4.5	26.8 ± 3.2	↑**
**Leucine**	49.1 ± 11.7	38 ± 7.6		32.2 ± 10.9	40 ± 2.8		18.2 ± 0.9	24.9 ± 5.8	↑*
**Lysine**	42.3 ± 15	13.6 ± 9.4	↓**	40.6 ± 6.7	24.4 ± 12.7	↓*	17.1 ± 13.8	62.1 ± 20.7	↑**
**Methionine**	65.6 ± 21.4	39.1 ± 27.6		21.3 ± 15.5	29.1 ± 19.4		13.1 ± 6.5	21.2 ± 8.9	
**Ornithine**	53.2 ± 30.1	28.8 ± 26.6		22.2 ± 14	40.7 ± 24		10.3 ± 4	19.6 ± 12.3	
**Phenylalanine**	51.8 ± 5.2	25.9 ± 11.9	↓**	33.4 ± 2.6	34.1 ± 9.2		15.6 ± 3.7	36.1 ± 3.7	↑***
**Proline**	50.2 ± 0.7	33.3 ± 4.6	↓***	29.9 ± 2.7	38.5 ± 2	↑***	20.5 ± 2.4	24.8 ± 4.3	
**Serine**	52 ± 3.2	32.7 ± 8.9	↓**	31.9 ± 6	41 ± 8.9		19.2 ± 1.7	26.3 ± 4.2	↑**
**Threonine**	33.4 ± 13.2	35.1 ± 4.6		40.3 ± 6.4	40.2 ± 2.3		26.3 ± 7.6	26.7 ± 2	
**Tryptophan**	33.5 ± 0.3	24.7 ± 13.3		38.2 ± 6.5	24.7 ± 13.3		33 ± 4.6	50.6 ± 26.7	
**Tyrosine**	37.2 ± 5.7	16.1 ± 13.3	↓**	33.4 ± 0.1	16.1 ± 13.3	↓*	23.9 ± 11.2	67.8 ± 26.7	↑**
**Valine**	43.2 ± 9.3	33.4 ± 8		33.1 ± 4.6	40.8 ± 2.6	↑*	20.7 ± 3.4	26 ± 3.4	↑*
**Putrescine**	52 ± 5	27.6 ± 10.8	↓***	33.8 ± 1.6	38.5 ± 8		15.8 ± 3.2	33.9 ± 6.5	↑***
**Spermidine**	33.3 ± 0	33.3 ± 0		33.3 ± 0	33.3 ± 0		33.3 ± 0	33.3 ± 0	

Table SII: Relative Distribution (Mean values ± Standard Deviation) of Rsch before and after cold acclimation. Asterisks indicate significant changes during acclimation per compartment (ANOVA & Tukey, * p<0.05; ** p<0.01; *** p<0.001) arrows (↑; ↓) indicate direction during acclimation.

Rsch [%]	Chloroplast			Cytosol			Vacuole		
Metabolite	Non-Acc	Acc	p	Non-Acc	Acc	p	Non-Acc	Acc	p
Fructose	23.5 ± 9.8	18.9 ± 8.9		28.3 ± 1.9	25.1 ± 6.1		52.6 ± 7	56 ± 13.5	
Galactinol	45.4 ± 11.6	33.1 ± 17.7		38.6 ± 9.9	39.3 ± 13.8		13.7 ± 4.9	27.6 ± 24.1	
Glucose	19.3 ± 10.7	18.2 ± 10.4		25 ± 5.9	23.9 ± 4.1		55.7 ± 16	57.9 ± 11.6	
Melibiose	19.3 ± 5.4	22.1 ± 17.4		24.4 ± 7	23.5 ± 14		56.2 ± 10.1	54.4 ± 30.5	
myo-Inositol	53.6 ± 8.1	37 ± 11.9	↓*	38.5 ± 11.2	46.7 ± 11.4		12.5 ± 7.6	16.3 ± 13	
Raffinose	44.9 ± 4.6	33.2 ± 18		40.5 ± 7.4	37.3 ± 15.6		17.6 ± 8.4	29.6 ± 26.7	
Sucrose	44.8 ± 11	33 ± 8.6	↓*	42 ± 7.4	46.2 ± 11.8		13.2 ± 7.7	20.8 ± 14.1	
Threitol	22.1 ± 11	28.6 ± 16		25.6 ± 7.9	28.3 ± 5.2		52.3 ± 17.5	43.1 ± 20.1	
2-Oxoglutarate	38.6 ± 9.3	32.4 ± 6.1		32.1 ± 5.9	43.6 ± 10.3	↑*	29.3 ± 9.8	23.9 ± 12.1	
Citrate	22.2 ± 1.2	20.8 ± 5.8		29.9 ± 9.6	35.7 ± 13.7		48.1 ± 12.9	43.5 ± 17.5	
Fumarate	15.2 ± 6.9	22.8 ± 9.4		20.6 ± 4.7	22.4 ± 5.1		61.7 ± 14.6	50.8 ± 20.5	
Gluconate	22 ± 11	30.2 ± 8.4		27.9 ± 10.4	37.2 ± 7.8		50.1 ± 20.6	34.2 ± 2.4	
Malate	19.7 ± 5	20.5 ± 4.9		30.6 ± 10.8	35.3 ± 12.9		49.7 ± 14.9	42.2 ± 19.1	
Oxaloacetate	30.7 ± 7.8	34.4 ± 14.8		30.2 ± 14	31.1 ± 3.3		31.3 ± 19.4	32.5 ± 16.3	
Pyruvate	28.9 ± 6.3	30 ± 5		26.4 ± 7.7	33.3 ± 0		42 ± 4.8	34 ± 5.2	↓*
Succinate	22.9 ± 12.6	21.4 ± 3.9		26.7 ± 7.8	28.9 ± 11.3		50.4 ± 19	53.4 ± 7.5	
Threonate	49.2 ± 9.2	39.9 ± 9.1		40.4 ± 7.6	43.5 ± 9.7		10.3 ± 5.3	16.6 ± 12.4	
Alanine	41.9 ± 10.8	39.6 ± 12.2		40 ± 8.3	43.8 ± 11.2		18.1 ± 6.8	16.6 ± 12.7	
Asparagine	50.8 ± 10.5	41.8 ± 11.3		37.3 ± 6.3	46.8 ± 13.5		12 ± 6	11.4 ± 11.6	
Aspartate	44.7 ± 8.9	42.8 ± 12.9		38 ± 6.1	42.5 ± 9.7		17.2 ± 7.6	14.7 ± 12.4	
Glutamate	48.4 ± 11	40.8 ± 9.3		37.9 ± 5.1	43.1 ± 7.4		11.5 ± 4.4	16 ± 10.9	
Glutamine	50.3 ± 9.7	32.9 ± 13.2	↓*	36.7 ± 5.4	39.9 ± 11.4		13 ± 6.7	27.2 ± 18.4	
Glycine	34.9 ± 12.7	25.2 ± 3		40.1 ± 6.3	42.8 ± 8.6		20.3 ± 7.9	32 ± 9.8	↑*
Isoleucine	42.4 ± 12.4	37.8 ± 11.8		39.3 ± 6.8	43.2 ± 11.5		15.7 ± 3.8	19 ± 11.9	
Leucine	44.1 ± 11.4	42.5 ± 12.5		40.6 ± 8.1	42.3 ± 8.7		15.3 ± 7.4	15.2 ± 11.6	
Lysine	41.5 ± 11.8	21.2 ± 8.3	↓**	40.9 ± 5.8	25.5 ± 13.3	↓*	17.7 ± 9.9	53.3 ± 21.1	↑**
Methionine	50.2 ± 9.2	41.9 ± 9.8		32.8 ± 5	41.3 ± 12.1		17 ± 7.2	16.8 ± 11.3	
Ornithine	47.9 ± 9.9	34.4 ± 5.1	↓*	38.6 ± 9	41.6 ± 10.6		13.5 ± 6.9	20.3 ± 13.4	
Phenylalanine	46.3 ± 11.5	39 ± 10.6		39.5 ± 8.1	40.1 ± 5.9		14.2 ± 6.1	15.2 ± 4.2	
Proline	44.6 ± 13.9	36.1 ± 10.2		38 ± 7.3	44.7 ± 11.5		17.4 ± 12.2	19.2 ± 16.1	
Serine	46.4 ± 11.8	35.7 ± 10.4		39.8 ± 6.7	46 ± 13.2		13.8 ± 7.5	18.3 ± 16.3	
Threonine	45.4 ± 13.2	37.6 ± 13		39.5 ± 7.6	45.6 ± 9.4		12.8 ± 3.3	12.9 ± 5.3	
Tryptophan	31.9 ± 7.9	28 ± 7		38.6 ± 9.4	34.8 ± 11.1		29.5 ± 13.6	37.2 ± 17.2	
Tyrosine	30.8 ± 18	16.3 ± 9		29.7 ± 9	23.2 ± 3.7		39.4 ± 23.1	65.8 ± 11.7	↑*
Valine	42.8 ± 11.4	39.9 ± 8.5		40.7 ± 6.7	44.1 ± 8.7		16.5 ± 6.6	16 ± 11.1	
Putrescine	47.6 ± 8.3	41.3 ± 6.5		42 ± 7.2	46.4 ± 9.2		10.4 ± 4.6	14.4 ± 3.2	
Spermidine	46.7 ± 10.1	39 ± 8.4		42.6 ± 7.6	33.7 ± 5.3	↓*	10.7 ± 5.6	27.3 ± 11.5	↑**
