# Supplementary material for: A Benchtop Fractionation Procedure for Subcellular Analysis of the Plant Metabolome
Source: Front Plant Sci. 2016 Dec 22;7:1912. doi: 10.3389/fpls.2016.01912 (PMC5177628; doi:10.3389/fpls.2016.01912)
Supplement: Supplementary Data 5 — Absolute compartmental distribution of metabolites in Cvi and Rsch. [file DataSheet5.PDF]

Supplement 5 – Absolute Amount of metabolites in Cvi and Rsch per compartment

Table SIII: Absolute Amount of Cvi before and after cold acclimation. Asterisks indicate significant changes during acclimation per compartment (ANOVA & Tukey, * p<0.05; ** p<0.01; *** p<0.001) arrows (↑;↓) indicate direction during acclimation.

Cvi [nmol/gFW]	Chloroplast			Cytosol			Vacuole		
**Metabolite**	**Non Acc**	**Acc**	**p**	**Non Acc**	**Acc**	**p**	**Non Acc**	**Acc**	**p**
**Fructose**	22.8 ± 6.9	607.4 ± 223.1	↑***	32.5 ± 2.2	902 ± 144.5	↑***	42.9 ± 9.9	1517.9 ± 239.2	↑***
**Galactinol**	14.9 ± 6.8	16.1 ± 3.8		21.3 ± 3.9	32.1 ± 1.5	↑***	29.8 ± 13.6	32.2 ± 4.6	
**Glucose**	101.3 ± 15.9	1741.1 ± 726.1	↑***	131.6 ± 28.7	2633.4 ± 545.2	↑***	215.4 ± 35.5	4432.6 ± 720.1	↑***
**Melibiose**	14.1 ± 1.4	13.5 ± 0		14.7 ± 0	13.5 ± 0	↓***	15.2 ± 1.1	13.5 ± 0	↓**
**myo-Inositol**	183.3 ± 19.8	1173.6 ± 289.5	↑***	130.8 ± 8	1268.6 ± 73	↑***	66.8 ± 3.3	836.4 ± 108.3	↑***
**Raffinose**	15.8 ± 9.8	20.9 ± 10.6		24.5 ± 1.5	29.9 ± 21.7		21.6 ± 10.4	62.2 ± 29	↑*
**Sucrose**	603.9 ± 133.2	2192.6 ± 548.1	↑***	850.7 ± 62.5	2955.5 ± 560.3	↑***	566.6 ± 82	2006.5 ± 306.7	↑***
**Threitol**	10.6 ± 5.7	2.9 ± 0	↓**	10.6 ± 5.7	2.9 ± 0	↓**	21.8 ± 11.5	33.4 ± 0	↑*
**2-Oxoglutarate**	12.7 ± 2.8	12.3 ± 2.3		14.5 ± 2.1	12.6 ± 1.8		10.3 ± 2.8	8.2 ± 1.2	
**Citrate**	17.6 ± 1.4	25.1 ± 6.7	↑*	39.3 ± 7.4	44.3 ± 5		44.2 ± 5	43.8 ± 2.9	
**Fumarate**	754.2 ± 113.2	4600.3 ± 1583.3	↑***	1328.9 ± 95.3	8276.7 ± 1366.6	↑***	1111.2 ± 22	9849.7 ± 1248.9	↑***
**Gluconate**	15.3 ± 0	14.4 ± 0	↓***	15.3 ± 0	14.4 ± 0	↓***	15.3 ± 0	14.4 ± 0	↓***
**Malate**	169.4 ± 30.5	538.2 ± 165.5	↑***	326.7 ± 6.2	809.3 ± 54.3	↑***	265.8 ± 16.2	699.7 ± 131.7	↑***
**Oxaloacetate**	7.5 ± 5.2	11.1 ± 0		7.5 ± 5.2	11.1 ± 0		21.9 ± 10.5	11.1 ± 0	↓*
**Pyruvate**	3.8 ± 0.5	2.7 ± 0	↓***	4.2 ± 0.3	2.7 ± 0	↓***	4.2 ± 0.3	2.7 ± 0	↓***
**Succinate**	56.2 ± 5.5	13.4 ± 5.2	↓***	83.9 ± 3.7	19.9 ± 3.3	↓***	66.7 ± 11.1	24.6 ± 5.9	↓***
**Threonate**	44.8 ± 1.2	24.9 ± 3.2	↓***	25.5 ± 4.3	26.8 ± 4.1		13.2 ± 2.7	16.9 ± 3.5	
**Alanine**	60.5 ± 11.7	40.8 ± 9.8	**	50.1 ± 10.6	43.2 ± 9.3		27.2 ± 1.7	29.8 ± 3.1	
**Asparagine**	28.2 ± 20.1	19.8 ± 12.9		8.9 ± 6.8	13.1 ± 8		6.5 ± 3.1	12 ± 14.6	
**Aspartate**	48 ± 2.9	79 ± 8	↑***	26 ± 4.8	45.6 ± 4.9	↑***	16.3 ± 4.1	33.8 ± 7.4	↑***
**Glutamate**	383.6 ± 11.6	104.2 ± 14.8	↓***	196.1 ± 39.4	63.7 ± 4.6	↓***	129.2 ± 10.1	46.7 ± 12.8	↓***
**Glutamine**	265.9 ± 18.5	138.1 ± 40.5	↓***	135.7 ± 29.9	179.2 ± 25.5	↑*	69.6 ± 14.3	239.4 ± 25	↑***
**Glycine**	23.9 ± 4	32.3 ± 8.7		29.8 ± 2.7	50.4 ± 5.4	↑***	16.9 ± 4.5	61.7 ± 12.4	↑***
**Isoleucine**	18.2 ± 4.1	13.3 ± 2.3	↓*	11.1 ± 1.9	14.4 ± 0.7	↑**	6.5 ± 1.7	9.6 ± 1.1	↑**
**Leucine**	17.4 ± 4.1	12.6 ± 2.5	↓*	11.4 ± 3.9	13.3 ± 0.9		6.4 ± 0.3	8.3 ± 1.9	
**Lysine**	12.9 ± 4.6	3.2 ± 2.2	↓**	12.3 ± 2	5.8 ± 3	↓**	5.2 ± 4.2	14.7 ± 4.9	↑*
**Methionine**	13.5 ± 4.4	7.3 ± 5.1		4.4 ± 3.2	5.4 ± 3.6		2.7 ± 1.3	3.9 ± 1.7	
**Ornithine**	11.8 ± 6.7	6.2 ± 5.7		4.9 ± 3.1	8.7 ± 5.2		2.3 ± 0.9	4.2 ± 2.7	↑**
**Phenylalanine**	28.5 ± 2.9	9.1 ± 4.2	↓***	18.4 ± 1.5	11.9 ± 3.2	↓**	8.6 ± 2.1	12.6 ± 1.3	↑**
**Proline**	30.9 ± 0.4	236.4 ± 32.7	↑***	18.4 ± 1.6	273.7 ± 14.4	↑***	12.6 ± 1.5	176.6 ± 30.7	↑***
**Serine**	121.3 ± 7.5	67.6 ± 18.4	↓***	74.6 ± 14	84.9 ± 18.5		44.7 ± 4	54.6 ± 8.8	↑*
**Threonine**	43.5 ± 17.2	66.9 ± 8.8	↑*	52.5 ± 8.3	76.7 ± 4.3	↑***	34.1 ± 9.9	50.8 ± 3.8	↑**
**Tryptophan**	8.6 ± 0.1	5.9 ± 3.2		9.9 ± 1.7	5.9 ± 3.2	↓*	8.5 ± 1.2	12 ± 6.3	
**Tyrosine**	10.2 ± 1.6	4.6 ± 3.8	↓*	9.1 ± 0	4.6 ± 3.8		6.5 ± 3.1	19.5 ± 7.7	↑**
**Valine**	30.1 ± 6.4	22.2 ± 5.3	↓*	23.1 ± 3.2	27.1 ± 1.7		14.4 ± 2.4	17.3 ± 2.3	
**Putrescine**	1 ± 0.1	0.9 ± 0.3		0.7 ± 0	1.2 ± 0.3	↑***	0.3 ± 0.1	1.1 ± 0.2	↑***
**Spermidine**	27 ± 0	25.2 ± 0	↓***	27 ± 0	25.2 ± 0	↓***	27 ± 0	25.2 ± 0	↓***

Table SIV: Absolute Amount of Rsch before and after cold acclimation. Asterisks indicate significant changes during acclimation per compartment (ANOVA & Tukey, * $p<0.05$; ** $p<0.01$; *** $p<0.001$) arrows (↑; ↓) indicate direction during acclimation.

**Rsch[nmol/gFW]**	**Chloroplast**			**Cytosol**			**Vacuole**		
**Metabolite**	Non Acc	Acc ± SD	p	Non Acc	Acc	p	Non Acc	Acc	p
**Fructose**	43.1 ± 17.9	3494.6 ± 1636.9	↑***	51.9 ± 3.5	4646.1 ± 1130.7	↑***	96.3 ± 12.8	10342.1 ± 2493.3	↑***
**Galactinol**	31.1 ± 8	68.7 ± 36.7	↑*	26.5 ± 6.8	81.6 ± 28.7	↑***	9.4 ± 3.4	57.3 ± 50.1	↑*
**Glucose**	236.1 ± 131	4881.5 ± 2779.4	↑***	305.3 ± 72.4	6417 ± 1102.8	↑***	679.9 ± 195.7	15510.7 ± 3103	↑***
**Melibiose**	10.8 ± 3	13.5 ± 10.7		13.7 ± 3.9	14.4 ± 8.6		31.5 ± 5.6	33.3 ± 18.7	
**myo-Inositol**	262.2 ± 39.7	1266 ± 407.3	↑***	188.3 ± 54.9	1597.7 ± 391.2	↑***	61.1 ± 37.4	556 ± 445.5	↑**
**Raffinose**	52.9 ± 5.4	4644.9 ± 2518.6	↑***	47.6 ± 8.8	5225.1 ± 2183.5	↑***	20.7 ± 9.9	4140 ± 3746.1	↑*
**Sucrose**	714.9 ± 175.7	6271 ± 1633.6	↑***	670.2 ± 117.9	8767.4 ± 2232.4	↑***	210.1 ± 122.6	3951.4 ± 2673.2	↑**
**Threitol**	11.8 ± 5.9	15.1 ± 8.5		13.8 ± 4.2	15 ± 2.8		28.1 ± 9.4	22.8 ± 10.6	
**2-Oxoglutarate**	17.8 ± 4.3	14.7 ± 2.8		14.8 ± 2.7	19.8 ± 4.7	↑*	13.5 ± 4.5	10.8 ± 5.5	
**Citrate**	19.1 ± 1.1	116.5 ± 32.3	↑***	25.8 ± 8.3	200.4 ± 77	↑***	41.5 ± 11.1	243.7 ± 98	↑***
**Fumarate**	1147.4 ± 523.1	9324.6 ± 3825.2	↑***	1549.5 ± 350.9	9164.7 ± 2081.8	↑***	4651.9 ± 1101.4	20760.3 ± 8365.9	↑***
**Gluconate**	11 ± 5.5	16.2 ± 4.5		14 ± 5.2	19.9 ± 4.2	↑*	25.1 ± 10.3	18.3 ± 1.3	
**Malate**	205.1 ± 51.7	1751.6 ± 421.2	↑***	318 ± 112.8	3017.1 ± 1100.7	↑***	516.6 ± 155.3	3615.7 ± 1634.7	↑***
**Oxaloacetate**	14.4 ± 3.7	16.3 ± 7		14.2 ± 6.5	14.8 ± 1.6		14.7 ± 9.1	15.4 ± 7.7	
**Pyruvate**	4.1 ± 0.9	5.3 ± 0.9	↑*	3.7 ± 1.1	5.9 ± 0	↑**	5.9 ± 0.7	6 ± 0.9	
**Succinate**	74.4 ± 40.8	129.3 ± 23.7	↑*	86.6 ± 25.2	174.8 ± 68.5	↑**	163.5 ± 61.7	322.5 ± 45.5	↑***
**Threonate**	54.7 ± 10.3	114.4 ± 26.2	↑***	45 ± 8.5	124.8 ± 27.9	↑***	11.5 ± 5.9	47.7 ± 35.6	↑**
**Alanine**	50.1 ± 12.9	73.6 ± 22.7	↑*	47.9 ± 10	81.6 ± 20.8	↑**	21.7 ± 8.1	30.9 ± 23.6	
**Asparagine**	29.3 ± 6.1	23.5 ± 6.3		21.5 ± 3.6	26.3 ± 7.6		6.9 ± 3.4	6.4 ± 6.5	
**Aspartate**	36.4 ± 7.2	118.3 ± 35.6	↑***	31 ± 5	117.4 ± 26.8	↑***	14 ± 6.2	40.5 ± 34.3	↑*
**Glutamate**	302.4 ± 68.6	497.4 ± 113.1	↑**	236.8 ± 31.6	525.4 ± 90.5	↑***	71.6 ± 27.6	195.1 ± 133.3	↑**
**Glutamine**	256.4 ± 49.6	446.6 ± 179.9	↑*	187.3 ± 27.4	542.9 ± 154.7	↑***	66.4 ± 34.4	369.6 ± 250.6	↑**
**Glycine**	18.1 ± 6.6	26.8 ± 3.2	↑**	20.8 ± 3.3	45.6 ± 9.2	↑***	10.5 ± 4.1	34 ± 10.4	↑***
**Isoleucine**	12.6 ± 3.7	17.5 ± 5.5		11.7 ± 2	20 ± 5.3	↑**	4.7 ± 1.1	8.8 ± 5.5	
**Leucine**	12 ± 3.1	15.3 ± 4.5		11.1 ± 2.2	15.2 ± 3.1	↑*	4.2 ± 2	5.5 ± 4.1	
**Lysine**	15.3 ± 4.3	6.6 ± 2.6	↓***	15.1 ± 2.1	7.9 ± 4.1	↓**	6.5 ± 3.6	16.5 ± 6.5	↑**
**Methionine**	12.9 ± 2.4	10.8 ± 2.5		8.4 ± 1.3	10.7 ± 3.1		4.4 ± 1.8	4.4 ± 2.9	
**Ornithine**	13.2 ± 2.7	10.6 ± 1.6		10.6 ± 2.5	12.8 ± 3.3		3.7 ± 1.9	6.2 ± 4.1	
**Phenylalanine**	17 ± 4.2	14.4 ± 3.9		14.5 ± 3	14.8 ± 2.2		5.2 ± 2.2	5.6 ± 1.5	
**Proline**	20 ± 6.2	829.5 ± 233.6	↑***	17 ± 3.3	1027.4 ± 264.8	↑***	7.8 ± 5.5	442.5 ± 369.2	↑**
**Serine**	202.6 ± 51.7	200.2 ± 58.1		173.6 ± 29.3	257.7 ± 73.8	↑*	60.3 ± 32.8	102.7 ± 91.2	
**Threonine**	55.1 ± 16	94.7 ± 32.8	↑*	47.9 ± 9.2	114.9 ± 23.7	↑***	15.5 ± 4	32.5 ± 13.2	↑*
**Tryptophan**	10.2 ± 2.5	9.3 ± 2.3		12.4 ± 3	11.6 ± 3.7		9.4 ± 4.4	12.4 ± 5.7	
**Tyrosine**	10 ± 5.8	8 ± 4.4		9.6 ± 2.9	11.3 ± 1.8		12.7 ± 7.5	32.2 ± 5.7	↑***
**Valine**	17.7 ± 4.7	28 ± 6	↑**	16.8 ± 2.8	30.9 ± 6.1	↑***	6.8 ± 2.7	11.2 ± 7.8	↑*
**Putrescine**	1.1 ± 0.2	1.5 ± 0.2	↑**	1 ± 0.2	1.7 ± 0.3	↑***	0.2 ± 0.1	0.5 ± 0.1	↑***
**Spermidine**	47.6 ± 10.3	38.8 ± 8.4		43.5 ± 7.7	33.6 ± 5.3	↓*	10.9 ± 5.7	27.2 ± 11.4	↑**
